# Carbon quantum dot incorporated AV/carboxymethyl cellulose hydrogel for enhanced wound healing

**DOI:** 10.1039/d6ra04173f

**Published:** 2026-09-24

**Authors:** K. Bhagyashri, Marvaan M. S., Samantha Raj Sah, Balashanmugam Panneerselvam, Devanand Venkatasubbu G.

**Affiliations:** a Department of Physics and Nanotechnology, SRM Institute of Science and Technology Kattankulathur Chengalpattu District Tamil Nadu 603203 India devanang@srmist.edu.in +91-9486456735; b Avigen Biotech Pvt Ltd Chennai Tamil Nadu India

## Abstract

Carbon quantum dots (CQDs), a novel class of nanomaterials, have received significant attention due to their unique properties, including excellent biocompatibility, low toxicity, and strong fluorescence. In this study, CQDs were integrated into a hybrid hydrogel matrix composed of carboxymethyl cellulose (CMC) and the bioactive components of aloe vera (AV) to leverage their synergistic wound healing and antimicrobial potential. AV, a renowned medicinal plant, has been traditionally used for wound healing. CMC, a versatile polymer, has excellent biocompatibility and moisture retention capabilities. The combination of AV, CMC, CQD and quercetin in a hydrogel formulation presents a promising approach for enhancing wound healing. In this study, we have synthesized CQDs and characterized them with X-ray diffraction (XRD), fourier-transform infrared spectroscopy (FTIR), photoluminescence (PL), ultraviolet-visible spectroscopy (UV), transmission electron microscopy (TEM), energy dispersive X-ray analysis (EDAX) and RAMAN spectroscopy. CQDs show very good antimicrobial properties against wound pathogens such as *Escherichia coli*, *Enterococcus faecalis*, *Staphylococcus aureus*, and *Pseudomonas aeruginosa*, studied through well diffusion, colony-forming unit (CFU), growth curve, biofilm assay, reactive oxygen species (ROS), cell membrane integrity, protein leakage, extracellular polymeric substances (EPS), and lipid peroxidation assessing its ability to inhibit infections in wounds. The synthesized hydrogels were evaluated *in vitro* for their morphology, antibacterial activity, swelling behavior, degradation profile, and rheological properties. The AV/CMC/CQD/QUECERTIN hydrogels show enhanced wound healing in *in vivo* wound healing experiments on albino Wistar rats with 89% wound closure on day 13 compared to the control which has only 58% wound closure.

## Abstract 2

Carbon quantum dots (CQDs), a novel class of nanomaterials, have received significant attention due to their unique properties, including excellent biocompatibility, low toxicity, and strong fluorescence.

## Graphical abstract

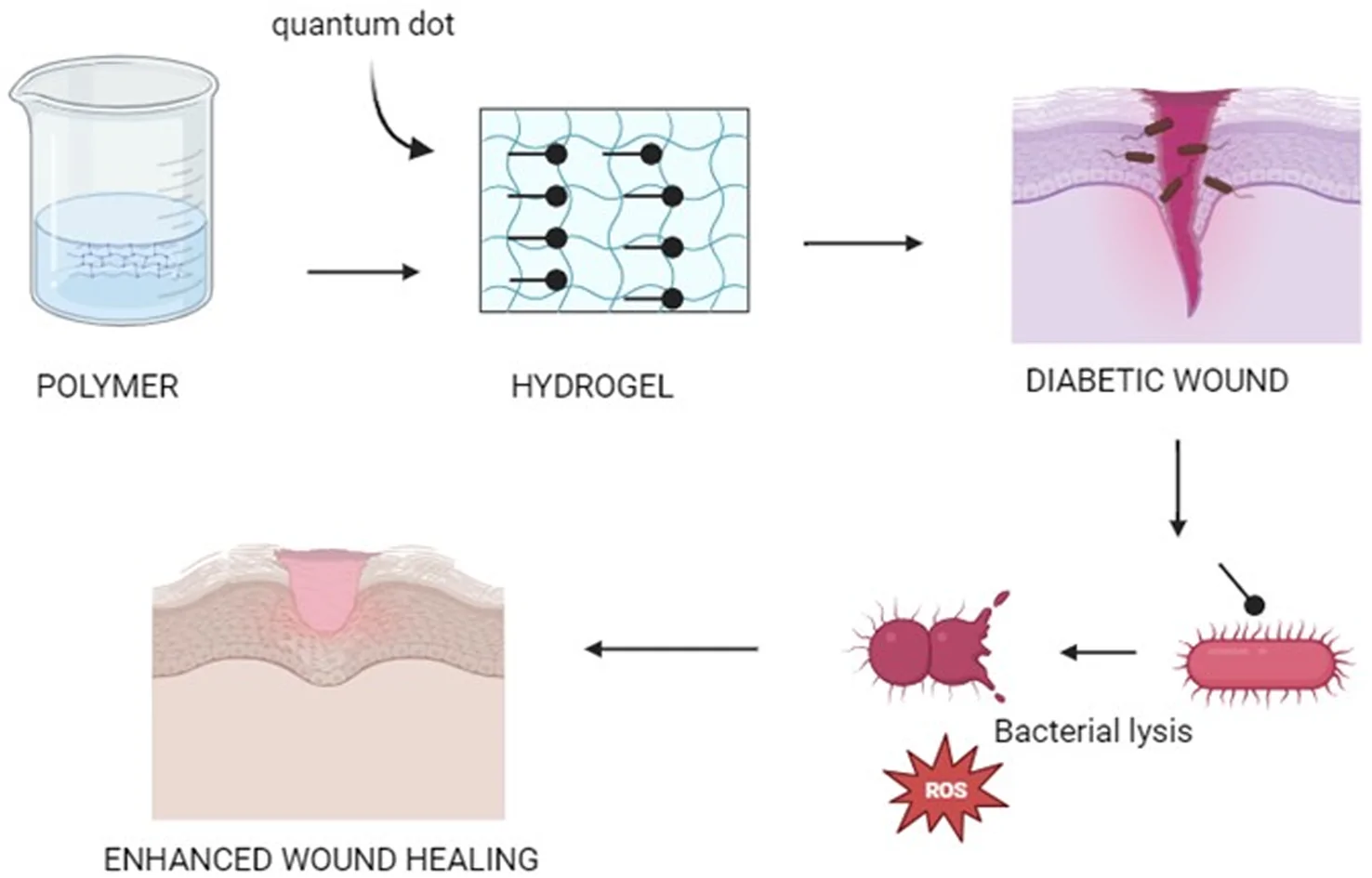

## 1 Introduction

The skin, acting as the body's largest organ, functions as a vital protective barrier that shields internal tissues from external hazards such as pathogens, harmful chemicals, ultraviolet radiation, and physical trauma.^1^ When this barrier is disrupted by injury, the body activates a complex wound healing process that progresses through four interrelated stages: hemostasis, inflammation, proliferation, and remodeling.^2^ Although the body has an intrinsic ability to repair wounds, this process can be hindered by factors such as chronic infections, diabetes, and aging, often resulting in delayed healing and a higher risk of secondary infections. There is, therefore, an urgent need for advanced wound dressings that go beyond mere physical protection by actively promoting tissue regeneration and preventing microbial colonization.

Over time, wound dressings have evolved from basic materials like gauze to sophisticated systems designed to interact dynamically with the wound environment. An ideal wound dressing should provide a moist environment, permit gas exchange, absorb exudates, prevent infection, and remain both biocompatible and biodegradable.^3^ Among the various materials developed for this purpose, hydrogels have attracted considerable interest. These hydrophilic, three-dimensional polymer structures can absorb substantial quantities of water or biological fluids while preserving their structural stability.^4^ Their high moisture content, softness, and flexibility closely resemble natural tissue, making them highly suitable for biomedical use. Furthermore, hydrogels can be engineered to deliver bioactive substances such as drugs, growth factors, and antimicrobial agents, enabling the creation of multifunctional wound care systems.^5^

AV, a succulent plant belonging to the Liliaceae family, is a promising natural source for hydrogel fabrication. Its gel contains numerous bioactive compounds including polysaccharides like acemannan, vitamins, enzymes, and antioxidants which possess anti-inflammatory and antimicrobial capabilities.^6^ These elements aid in stimulating fibroblast growth, enhancing collagen production, and promoting angiogenesis all critical processes for efficient wound healing.^7^ Hydrogels formulated with AV are naturally biocompatible and non-toxic, making them highly suitable for clinical wound care applications.

Despite these advantages, AV hydrogels can be further enhanced by incorporating functional nanomaterials. CQDs are a notable category of nanomaterials; these carbon based particles, generally less than 10 nanometers in size, possess exceptional properties including intense photoluminescence, high surface area, and low toxicity.^8^ In the context of wound healing, CQDs have demonstrated key aspects of tissue regeneration, such as antimicrobial effects, antioxidant activity, and the ability to promote cell migration.^9^ Integrating CQDs into AV hydrogel produces a synergistic effect. CQDs enhance the antibacterial activity of the hydrogel, while their antioxidant properties reduce oxidative stress at the wound site, facilitating faster regeneration.^10^ Recent advancements in nanotechnology have positioned CQDs as a superior alternative to traditional metal-based nanoparticles due to their exceptional biocompatibility, tunable surface chemistry, and potent antimicrobial properties.^11^ Unlike conventional antibiotics, CQDs exert a multi-target inhibitory effect *via* ROS generation and physical membrane disruption, which significantly mitigates the risk of bacterial resistance.^12^ CMC, a polymer derived from cellulose, is valued for its safety and ability to naturally break down. It functions as an effective binder and moisture regulator, forming protective films that support the integrity of the dressing.^13^ While AV/CQD composite hydrogels exhibit significant potential, in-depth studies are needed to evaluate their mechanical strength, swelling behavior, and drug release dynamics.^14^ Furthermore, the wound care has shifted toward ‘smart’ nanocomposites where CQDs are doped into biopolymer matrices like CMC or bacterial cellulose. These hybrid hydrogels leverage the high porosity and moisture retention of cellulose derivatives while utilizing the CQDs as functional dopants that enhance structural integrity and provide sustained therapeutic release. Such doped systems have shown remarkable efficacy in inhibiting biofilm-forming pathogens, making them highly relevant for the development of next-generation dressings that bridge the gap between traditional scaffolds and advanced carbon-based therapeutics.^15^ Additionally, quercetin has been incorporated for its potential in treating wounds.^16^ It exhibits antioxidant properties and modulates key signaling pathways, such as PI3K/Akt, to promote faster closure.^17^

While individual biomolecules such as AV, CMC, CQDs, and quercetin have been independently explored for wound care, integrating them into a cohesive therapeutic hydrogel presents distinct interfacial and kinetic advantages. Prior CQD-based hydrogels often suffer from rapid burst release of nanoparticles or rigid mechanical behavior that poorly conforms to irregular tissue contours. In this study, we leverage the natural polysaccharide network of CMC and AV to construct a non-covalently crosslinked matrix, where CQDs serve a dual role: as structural nano-reinforcements and as active functional agents. By combining CQD mediated antibacterial activity with the potent anti-inflammatory effects of loaded quercetin, this system addresses the multi-factorial challenges of exudate management, bacterial contamination, and oxidative stress in a single matrix. Rather than proposing a completely novel class of materials, this work provides a detailed investigation into optimizing the physical crosslinking, degradation kinetics, and synergistic bioactivity of a multi-component biopolymer system for enhanced tissue repair.

Recent studies (2023–2025) emphasize the potential of CQD based hydrogels for treating complex, non-healing wound beds. CQDs provide strong photoluminescence and photo-triggered antibacterial action, while natural bio-actives like quercetin reduce oxidative stress and inflammation. Combining CQDs and quercetin into a physical hydrogel network yields a multifunctional dressing that offers both structural support and dual therapeutic benefits.^18^

In this study, we hypothesize that incorporation of CQDs into AV/CMC hydrogel will synergistically enhance antimicrobial activity and accelerate wound healing in cutaneous wounds by promoting bacterial inhibition, keeping a moist wound environment, and supporting cellular proliferation and tissue regeneration. Our work introduces a design that moves beyond simple mixtures. The deep mechanistic insight lies in the coordinated constructive interaction of the four components: the CQDs function as the “active disruptor” of bacterial defense, while the AV/CMC matrix creates a microenvironment of quercetin for anti-inflammatory action. Our microwave assisted synthesis provides a faster, alternative to traditional hydrothermal methods, yielding CQDs with superior blue photoluminescence (432 nm). Furthermore, showing a superior therapeutic efficacy that advances the current multifunctional wound care.

## 2 Materials and methods

### 2.1. Materials

Citric acid (C_6_H_8_O_7_), ethylenediaminetetraacetic acid (EDTA, C_10_H_16_N_2_O_8_), (CMC; viscosity 400–800 cP; degree of substitution ≈ 0.7) with a reported viscosity average molecular weight (*M*_v_) of 250 kDa), glycerol (C_3_H_8_O_3_; M.W. 92.09 Da), dimethyl sulfoxide (DMSO, C_2_H_6_OS), ethanol (C_2_H_5_OH), crystal violet (C_25_H_30_ClN_3_), Triton X-100 (C_14_H_22_O(C_2_H_4_O)_*n*_), phosphate-buffered saline (PBS), sodium chloride (NaCl), phenol (C_6_H_5_OH), sulfuric acid (H_2_SO_4_), boric acid (H_3_BO_3_), carbazole (C_12_H_9_N), Bradford reagent, trichloroacetic acid (CCl_3_COOH), thiobarbituric acid (C_4_H_4_N_2_O_2_S), MTT solution (C_18_H_16_BrN_5_S), and chloroform (CHCl_3_) were obtained from Sisco Research Laboratories Pvt. Ltd (SRL, Chennai, India). Nutrient agar and nutrient broth were also procured from SRL. A dialysis membrane, deionized (DI) water, and pure AV gel were employed in the study. All chemicals and solvents were of analytical grade and used as received without further purification.

### 2.2. Synthesis of CQDs

Water soluble CQDs were synthesized using a top down microwave assisted synthesis. Initially, 1 gram of citric acid was dissipated in 25 mL of deionized (DI) water. Ethylenediamine was then added to the solution. The mixture was exposed to high power microwave irradiation for 8 min under open air conditions. During the process, the solution's color changed from colorless to pale yellow, then to strong yellow and eventually brown, indicating the successful formation of CQDs.^19^ The resulting CQDs were subsequently purified by dialyzing against distilled water for 48 h using a dialysis membrane with a molecular weight cut-off of 2 kilo Dalton. Finally, the solution was stored under optimal conditions.

### 2.3. Characterization

The crystalline phase and structural purity of the synthesized liquid CQDs were determined by XRD, over 2*θ* = 10°–80° by drop casting the liquid sample onto a zero-background substrate and drying under vacuum. Surface functional groups and chemical bonding were identified *via* FTIR, using an Attenuated Total Reflectance (ATR) module in the wavenumber range of 4000–400 cm^−1^. PL spectra and excitation-dependent emission profiles were recorded on a spectrofluorometer by loading the aqueous CQD solution into a 1 cm path-length quartz cuvette across excitation wavelengths ranging from 300–500 nm. The nanoscale morphology, particle size distribution, and lattice spacing were visualized using TEM, after drop-casting 5 µL of diluted CQD dispersion onto a carbon-coated copper grid and air-drying. Elemental mapping and qualitative composition were determined by EDX coupled to the electron microscope. Surface charge and colloidal stability were evaluated *via* Zeta potential analysis using a Zetasizer by diluting the liquid sample in ultrapure water (0.1 mg mL^−1^) and measuring Phase Analysis Light Scattering (PALS) at 25 °C. Graphitic disorder and structural defects (*I*_D_/*I*_G_ ratio) were analyzed using a micro-Raman spectrometer with a 532 nm laser source on a vacuum-dried drop-cast CQD film. Finally, optical absorption properties were recorded using a UV-Visible spectrophotometer between 200–800 nm using deionized (DI) water as a reference blank.

### 2.4. Fluorescence quantum yield

The fluorescence quantum yield (QY) of the synthesized CQDs is determined using a comparative method with a standard fluorophore. Quinine sulfate (*Φ* = 0.54 in 0.1 M H_2_SO_4_) is used as the reference. The CQD dispersion is diluted with distilled water to obtain an absorbance below 0.1 at the excitation wavelength, minimizing inner filter effects. The reference solution is prepared under identical absorbance conditions.

The quantum yield of CQDs is calculated using the comparative relation:

1 *Φ*_s_ = *Φ*_r_ × (*I*_s_/*I*_r_) × (*A*_r_/*A*_s_) × (*n*_s_^2^/*n*_r_^2^)

where *Φ* is the quantum yield, *I* is the integrated emission intensity, *A* is the absorbance at the excitation wavelength, and *n* is the refractive index of the solvent.

### 2.5. Minimum inhibitory concentration (MIC)

To determine the minimum inhibitory concentration (MIC) and percentage of growth inhibition of the synthesized CQDs, a broth microdilution assay was performed in accordance with standard guidelines. Bacterial cultures were treated with CQDs across a gradient of working concentrations (10, 12, 14, 16 mg mL^−1^) and incubated for 24 h at 37 °C. The experimental setup included a test group (culture + broth + CQDs), a negative control (broth + CQDs), a viability control (Vc, culture only), and a media blank (broth only). Following incubation, optical density (OD) was measured at 620 nm to quantify bacterial growth. The percentage of growth inhibition was calculated using the normalized formula:

2 Growth inhibition (%) = 1 − (OD_Test_ − OD_Neg_/OD_Vc_ − OD_Broth_) × 100

This calculation accounts for the inherent absorbance of the CQDs and the culture medium, ensuring an accurate representation of the nanomaterial's antimicrobial efficacy.

In compliance with standard microbiological definitions, the MIC was defined as the minimum concentration achieving complete growth suppression (90–100% inhibition), whereas lower concentrations exhibiting partial growth reduction were classified as sub-inhibitory concentrations (Sub-MIC).

### 2.6. Well diffusion

The antibacterial activity of CQD was analyzed against four distinct pathogenic clinical bacterial stains that are frequently detected on the surface of wounds. Two Gram-positive bacteria's namely *Staphylococcus aureus and Enterococcus faecalis* (ATCC stain 25923 and 29212) and two Gram-negative bacteria's including *Pseudomonas aeruginosa and Escherichia coli* (ATCC stain 27853 and 25922)^20^ were tested against CQDs in the Nutrient agar media. Subsequently, 100 µL of each subculture strain was evenly distributed onto Petri dishes using an L-rod. Wells, with a diameter of 6 mm, were then created in the medium using a well borer. CQD was diluted and added to the well into four distinct concentrations, 10 mg mL^−1^, 12 mg mL^−1^, 14 mg mL^−1^ and 16 mg ml^−^^21^. The Petri dishes were incubated for 24 h at 37 °C. The zone of inhibition was observed after 24 h of incubation and measured.

### 2.7. Growth curve

A growth curve microbial assay serves as a foundational method in microbiology for examining the growth patterns of microorganisms over time. This approach entails tracking the population density of bacteria, in a liquid culture at various intervals to construct a growth curve.^22^ Bacteria were cultured in Nutrient broth media supplemented with varying concentrations of synthesized samples 10 mg mL^−1^, 12 mg mL^−1^, 14 mg mL^−1^ and 16 mg mL^−1^. These cultures were incubated in a shaking incubator set at 180 rpm at 37 °C. Optical density was recorded using a UV-Spectrometer at 2 h intervals over 24 h period to observe the dynamics of bacterial growth.

The experiment was done in triplicate.

### 2.8. Colony count

A colony counting microbial assay, also known as a plate count or viable count assay, was a standard laboratory method used to determine the number of viable microorganisms. Nutrient broth cultures of *Escherichia coli*, *Enterococcus faecalis*, *Staphylococcus aureus*, and *Pseudomonas aeruginosa* were standardized to a 0.5 McFarland standard, and an aliquot of 100 µL of each standardized suspension was used to inoculate the broth. Subsequently, 200 µL of CQDs at concentrations of 10, 12, 14, and 16 mg mL^−1^ and 10 mg was added and incubated at 37 °C for 24 h. After 24 h, samples were serially diluted to 10^6^, plated on agar, incubated at 37 °C for 24 h, and colonies were imaged.^23–25^

### 2.9. Biofilm disruption assay

Biofilm disruption was evaluated using a well plate method. Bacterial cultures were treated with CQD's at different concentrations (10 mg mL^−1^, 12 mg mL^−1^, 14 mg mL^−1^ and 16 mg mL^−1^) and incubated at 37 °C for 24 h. Following incubation, the wells were gently rinsed with phosphate buffered saline (PBS) and subsequently stained with 0.1% crystal violet (CV). Optical density (OD) measurements were taken at 595 nm using a UV-Vis spectrophotometer. All experiments were carried out in triplicate.^26^ The extent of biofilm disruption induced by CQD's was quantified using the biofilm disruption formula.

3 Biofilm disruption (%) = OD_(control)_ − OD_(test sample)_/OD_(control)_ × 100

### 2.10. Biofilm formations

Bacterial strains were grown on agar plates, and the plates were incubated for 24 h at 37 °C. After the incubation of CQDs they were spread to the plates, which were then further incubated for 3 h. To eliminate non-adherent bacteria, the plates were being rinsed thrice with saline and subsequently made to dry for about 30 min^27^. To evaluate biofilm formation while preventing background interference from dye absorbed by the agar, control plates (without biomass) were processed in parallel to subtract nonspecific staining, or alternative rinsing steps were optimized. The plates were stained with 0.5 (w/v) crystal violet for 20 min, thoroughly rinsed with PBS to remove residual unbound dye, and allowed to air dry. The retained dye was subsequently solubilized using 95% ethanol. The dimensions of the stained areas were determined using digital vernier caliper by measuring across perpendicular axes. High contrast between the stained target regions and the underlying agar permitted clear boundary identification. Uninoculated agar plates subjected to the same staining and rinsing protocols served as negative controls to verify that background dye retention did not interfere with boundary determination.

### 2.11. Reactive oxygen species

To assess the intracellular ROS generation induced by CQDs, a DCFDA (2′,7′-dichlorodihydrofluorescein diacetate) cellular assay was performed. Briefly, bacterial cultures (*E. faecalis, S. aureus, E. coli,* and *P. aeruginosa)* were incubated for 24 hours at 37 °C with varying concentrations of CQDs (10, 12, 14, and 16 mg mL^−1^), followed by centrifugation at 5000 rpm for 5 minutes to harvest the cells. The resulting pellets were washed and resuspended in 2 mL of Phosphate Buffered Saline (PBS) before being treated with 200 µL of DCFDA dye. The suspension was incubated in dark conditions for 20 minutes to allow for the oxidative conversion of the non-fluorescent dye into highly fluorescent 2′,7′- dichlorofluorescein (DCF). The fluorescence intensity was subsequently measured at an excitation wavelength of 485 nm using a spectrofluorometer, with a blank (PBS + DCFDA).

### 2.12. Cell membrane integrity

To evaluate the impact of CQDs on bacterial cell membrane integrity, the leakage of intracellular constituents was monitored *via* UV-Visible spectrophotometry. Following a 24-hours incubation of the bacteria (*E. faecalis*, *S. aureus*, *E. coli*, and *P. aeruginosa*) with CQDs at concentrations of 10, 12, 14, and 16 mg mL^−1^, the bacterial suspensions were centrifuged at 5000 rpm for 10 minutes to separate the cells. The resulting pellets were carefully washed with Phosphate Buffered Saline (PBS) to remove external contaminants without disturbing the cell mass, then resedimented through a secondary centrifugation cycle. After resuspending the final pellets in 2 mL of PBS, absorbance was measured at 260 nm. This measurement served as a direct indicator of membrane damage, with increased absorbance values reflecting greater compromise of the lipid bilayer in response to the nanomaterial treatment.

### 2.13. Exopolysaccharide assay (EPS)

The concentration of EPS was quantified using the phenol–sulfuric acid method.^28^ Biofilms formed by different bacterial strains were treated with CQD's. Following treatment, the samples were washed with 0.9% NaCl and vortexed briefly to extract the EPS solution. An equal volume of 5% phenol and concentrated H_2_SO_4_ (99.99%) was added to the solution, followed by incubation for 1 h. The mixture was then centrifuged at 5000 rpm for 5 min. The absorbance of the resulting supernatant was recorded at 490 nm. The experiment was performed in triplicate.

The EPS content was calculated with the formula given below.

4 EPS (%) = [(OD_C_ − OD_T_)/OD_C_] × 100

### 2.14. Protein leakage assay

Protein leakage was analyzed using Bradford assay.^29^ Bacterial cultures were grown with CQDs and without CQDs. At 5000 rpm the cultures were then centrifuged separately for about 10 at 5000 rpm. Then 2.5 mL of 1 : 4 diluted Bradford reagent and 375 µL sample of the supernatant was mixed. Then (OD) at 595 nm was being measured using a UV-Vis spectrophotometer after 20 min. The experiment was triplicated.

### 2.15. Lipid peroxidation assay

To evaluate lipid peroxidation, 200 µL of CQD's and 50 µL of 0.9% NaCl (w/v) were incubated with 100 µL of overnight bacterial culture at 37 °C for 1 h^30^. Following this, an equal volume of 10% trichloroacetic acid was added, and the mixture was incubated at room temperature for 20 min. The samples were then centrifuged at 10 000 rpm for 10 min, after which 600 µL of 1.0% Thio barbituric acid (TBA) was introduced. The reaction mixture was heated for 10 min, cooled to room temperature, and maintained for 3 h. Absorbance was measured at 532 nm to determine the extent of lipid peroxidation, with values compared against untreated controls. All experiments were performed in triplicate.

### 2.16. Extraction of aloevera

Mature leaves of AV (*Aloe barbadensis*) were manually harvested and carefully washed with fresh water and then by deionized (DI) water. The outer rind and yellow exudates were removed using a sterile knife to isolate the inner fillet. These fillets were then blended using a domestic blender to produce a uniform pulp. A 60 mL sample of the pulp was being centrifuged for 30 min at 3000 rpm to separate gel from surplus water.^31^ The supernatant was further filtered using Whatman filter paper to extract the pure gel. This gel was dried at 80 °C for 8 h, then finely ground using a porcelain mortar to obtain AV powder, which was stored in an airtight container away from light.^32^

### 2.17. Synthesis of hydrogels

The hydrogel matrices were synthesized *via* a physical crosslinking method utilizing an optimized concentration of 9 wt% CMC and 5 wt% AVP dissolved in deionized (DI) water under continuous magnetic stirring at 60 °C, supplemented with glycerol as a plasticizer to yield the pure base matrix (hydrogel A). The 9 wt% CMC loading was experimentally optimized, as concentrations 8 wt% produced an excessively watery, fluid-like state, 10 wt% resulted in an overly rigid, unworkable gel network. To prepare the nanomaterial-incorporated matrix (hydrogel B), liquid CQDs at a final concentration of 16 mg mL^−1^ were introduced dropwise into hydrogel A under vigorous agitation to achieve homogeneous dispersion. Subsequently, for the drug-loaded dual nanocomposite (hydrogel C), quercetin (16 mg mL^−1^) dissolved in DMSO was uniformly blended into hydrogel B under dark conditions. All three formulations (hydrogels A–C) formed homogeneous, highly viscous gel-like matrices that eliminate the need for casting into rigid pre-defined molds, allowing them to flexibly adapt and conform to complex, irregular wound topographies upon topical application.

### 2.18. Rheology

The quantitative mechanical and viscoelastic deformation properties of the hydrogel formulations (hydrogels A–C) were evaluated using a modular compact rheometer equipped with a 25 mm parallel-plate geometry (1.0 mm gap distance) at 37 °C. Continuous shear stress (*τ*) *versus* shear strain (*γ*) measurements were performed to quantify the mechanical response under controlled shear deformation. The shear modulus (*G*), representing quantitative matrix stiffness prior to structural yield, was calculated from the slope of the linear elastic region (Δ*τ*/Δ*γ*). In addition, the yield stress (*τ*_y_) and yield strain (*γ*_y_) were derived directly from the stress–strain profiles to identify the critical stress threshold required to induce gel flow and evaluate matrix resistance against physical deformation.

### 2.19. Injectability test for hydrogel

The hydrogels were initially prepared under sterile conditions and transferred into a clean syringe (ranging from 1 mL to 5 mL) fitted with an appropriate needle, of 21 G. Care was taken to avoid the formation of air bubbles. The syringe was kept upright, and consistent manual pressure was applied to the plunger to dispense the hydrogel.^7^ The injection process was qualitatively assessed by observing the smoothness of the flow, any instances of blockage, and the overall ease of hydrogel extrusion.

### 2.20. Inversion test for hydrogel

To evaluate the structural integrity and stability of the hydrogel, the inversion test was performed. 1.0 g of hydrogel was placed in a clean, transparent vial and securely sealed. The container was then inverted to assess whether the gel maintained its position without flowing.^33^ A hydrogel that remained stationary upon inversion was considered to have sufficient gel strength and stability. This test reflects the formulation's resistance to gravitational forces and its ability to retain its shape. The experiment was conducted in triplicate.

### 2.21. Spreadability of the hydrogel

The spreadability of the hydrogels were assessed to evaluate its ease of application and uniform distribution over a surface.^34^ 1.0 g of hydrogel (typically 100 mg) was placed between two glass plates (7 × 7 cm). A standard weight (usually 1000 g) is applied to the top plate for 60 s to facilitate spreading. After removing the weight, the diameter or area covered by the hydrogel was measured. Spreadability was expressed based on the extent of spreading, with a larger spread indicating better spreadability. The test was performed in triplicate.

The spreadability (*S*) can be calculated using the formula.

5 *S* = *M* × *L*/*T*

where *M* is the weight placed above the glass slide (g), *L* is the distance the hydrogel spreads (cm), and *T* is the time (s).

### 2.22. Swelling of hydrogels

The fluid uptake capacity of the hydrogels was quantified using the gravimetric swelling ratio. Dried hydrogel samples (approximately 1.0 g) were weighed (*W*_d_) and then immersed individually in 10 mL of Phosphate Buffered Saline (PBS) of pH 7.4 maintained at 37 °C. For every 2 h, samples were retrieved, gently blotted using filter paper to remove excess surface moisture, and reweighed (*W*_s_). This process continued until the equilibrium swelling weight was reached.^35^ The swelling ratio (SR) was calculated as:

6 SR = (*W*_s_ − *W*_d_)/*W*_d_ × 100

All measurements were performed in triplicate to ensure data reproducibility.

### 2.23. Degradation of hydrogels

The structural stability of the hydrogels were evaluated by monitoring mass loss over an extended period. Fully formed hydrogel samples (approximately 1.0 g), with their initial dry weight recorded (*W*_i_), were individually immersed in 10 mL of PBS of pH 7.4 solution and incubated at 37 °C. Samples were retrieved for every 2 h. After retrieval, the samples were thoroughly washed with deionized (DI) water, lyophilized or dried in a vacuum oven until a constant final dry weight (*W*_t_.) was achieved. The percentage of degradation was calculated using the standard formula: All data points were averaged from triplicate measurements.

7 Degradation (%) = (*W*_i_ − *W*_t_)/*W*_i_ × 100

### 2.24. Colony count of hydrogel

The antibacterial potency of the hydrogel matrices (hydrogel A, hydrogel B, and hydrogel C) against Gram negative (*Escherichia coli*, *Pseudomonas aeruginosa*) and Gram positive (*Enterococcus faecalis*, *Staphylococcus aureus*) bacteria was quantitatively evaluated using the standard agar plate serial dilution method. Briefly, bacterial suspensions exposed to hydrogel samples for 24 h at 37 °C were serially diluted in sterile PBS, (pH7.4) from 10^−1^ to 10^−5^. Aliquots (100 µL) from appropriate dilution tubes were spread evenly onto nutrient agar plates and incubated at 37 °C for 24 h. Dilution plates exhibiting between 30 and 300 distinct, non-overlapping colonies were selected for quantitative colony counting. The concentration of viable bacteria (CFU mL^−1^) and the percentage antibacterial efficacy (%) were calculated using eqn (1) and (2), respectively:

8 CFU mL^−1^ = (colony count (N) × dilution factor (DF)/volume plated (V, mL)

9 Antibacterial efficacy (%) = (CFU_control_ − CFU_treated_/CFU_control_) × 100

All assays were performed in triplicate (*n* = 3), and results are reported as mean ± standard deviation.

### 2.25. MTT assay

The biocompatibility of the developed hydrogels was assessed using the MTT assay on NIH3T3 fibroblast cells. Cells were seeded into 96-well plates at a density of 1 × 10^4^ cells per well and allowed to establish for 24 h, after which sterilized hydrogel specimens were introduced and co-incubated under standard culture conditions (37 °C, 5% CO_2_) for 72 h. Post-exposure, the medium was substituted with MTT solution (0.5 mg mL^−1^), and the cells were incubated for an additional 4 h to facilitate formazan crystal formation. The insoluble crystals were subsequently solubilized using DMSO, and absorbance was quantified at 620 nm. Cell viability was determined in comparison with untreated controls, and all experiments were conducted triplicate, with data reported as mean ± SEM to establish the biocompatibility of the hydrogels.^36^

### 2.26. Live dead assay

Seed cells in a sterile 24-well plate and allow them to reach the desired confluence. Treat cells with test samples and incubate for the required exposure period. Prepare an AO/PI staining solution containing 5 µg mL^−1^ acridine orange (AO) and 5 µg mL^−1^ propidium iodide (PI) in PBS. Remove medium, gently wash cells with PBS, and add 200 µL of the AO/PI solution to each well. Incubate for 5 minutes at room temperature in the dark. Wash once with PBS and observe immediately under a fluorescence microscope. Live cells appear green (AO), while dead or membrane-compromised cells fluoresce red (PI).^37^ All experiments are triplicated. Image-based quantitative cell counting was conducted using ImageJ software. For each group, live and dead cells were enumerated across three randomly chosen microscopic fields (*n* = 3). The cell viability percentage was calculated using eqn (10):

10 Cell viability(%) = (*N*_live_)/(*N*_live_ + *N*_dead_) × 100

### 2.27. *In vivo* animal studies

#### 2.27.1. Experimental animals

The wound healing procedure follows CPCSEA guidelines, evaluated and approved by The Institutional Animal Ethical Committee, SRM Institute of Science and Technology, India.The *in vivo* experiment was conducted over a continuous 13-days period using a total of 30 subjects (*n* = 30), which were divided into five experimental groups (*n* = 6 per group) weighing between 130–150 g. Animals were randomly allocated into experimental groups using a simple randomization approach to minimize selection bias. To eliminate social variables and ensure precise tracking of individual metabolic data, all animals were housed in individual isolation cages throughout the duration of the study. Each subject was maintained on a standardized bedding to ensure consistent environmental and sanitary conditions. Nutritional requirements were met through the daily provision of feed, and hydration was managed through a strict 24 h water exchange protocol. This daily replenishment of both food and water allowed for the accurate monitoring of consumption and the mitigation of daily water wastage, ensuring a controlled and hygienic environment for the duration of the 13-days protocol.^38^

#### 2.27.2. Wound model

Prior to wound induction, the dorsal region of each rat was carefully shaved using a sterile razor. A square area measuring 1.5 × 1.5 cm was then demarcated on the exposed skin, and the marked section was excised using a sterile surgical blade to create a full-thickness wound model.

#### 2.27.3. Treatment groups

*A priori* power analysis was performed to determine the appropriate sample size for the *in vivo* wound healing study. Based on preliminary data and effect sizes observed in similar biomaterial-based wound healing studies, a power calculation (power = 0.8, *α* = 0.05) indicated that a minimum of 5 animals per group would be sufficient to detect statistically significant differences in wound closure rates. The animals were caged into five groups, each comprising six rats, including one negative control, one positive control, and three treatment groups. The negative control group (G1) received no therapeutic intervention, with wounds simply covered using sterile surgical dressings. In the positive control group (G2), animals were treated every alternate day with a commercially available ointment, Hydra-Heal. The treatment groups (G3, G4, and G5) were administered with hydrogels A–C respectively, with 100 mg hydrogels applied daily. Wound dimensions were recorded on alternate days, and all animals were closely observed throughout the study period. On day 13, the rats were anesthetized and humanely sacrificed in compliance with institutional ethical guidelines. The wound measurement and image analysis have been performed in blinded manner, where the investigator assessing wound closure was unaware of group allocation.

### 2.28. Statistical analysis

All *in vitro* experiments were performed in triplicate (*n* = 3), while the *in vivo* studies were also conducted with six animals per group (*n* = 6). All data are expressed as mean ± standard error of the mean (SEM). Statistical evaluations were performed using GraphPad Prism 6.0. One-way ANOVA was applied to determine the effect of different concentration groups compared with the control group. A *p*-value of less than 0.05 was considered statistically significant with confident interval of 95%. Comparisons among multiple groups were carried out using one-way analysis of variance (ANOVA), followed by Tukey's multiple comparison post hoc test. Differences were considered statistically significant when *p* < 0.05.

## 3 Results and discussion

### 3.1. XRD, FTIR, PL

**Fig. 1:**
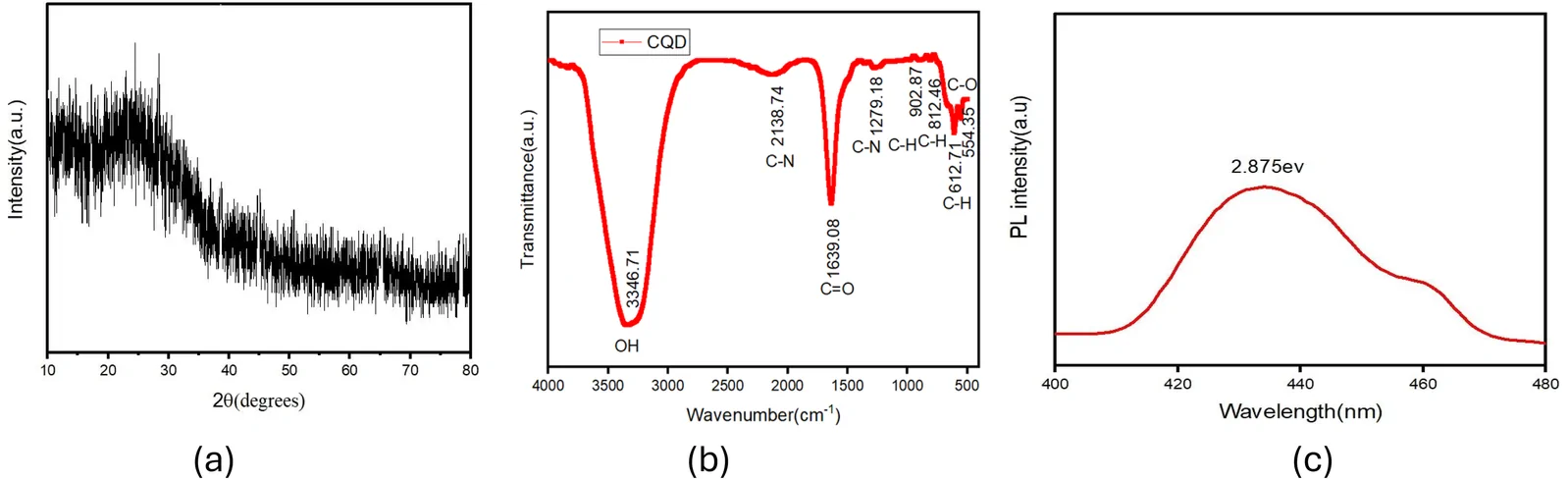
Structural and optical characterization of carbon quantum dots (CQDs): (a) XRD pattern showing the structural characteristics of the CQDs, (b) FTIR spectrum identifying the characteristic surface functional groups, and (c) PL spectrum demonstrating the optical emission behavior of the CQDs.

Fig. 1(a) shows the XRD pattern of CQD. XRD is a commonly employed method to determine whether a material is crystalline or amorphous by analyzing the diffraction patterns generated as X-rays interact with the atomic planes within the sample.^39^ In the case of CQDs, XRD was employed to evaluate their structural characteristics. The XRD pattern displayed a broad peak at 2*θ* = 24.5°, which is characteristic of amorphous carbon structures. This broad diffraction peak indicates the lack of long-range order in the CQDs, confirming their amorphous nature. Unlike crystalline materials that exhibit sharp and well defined peaks, the broadness of the observed peak is typical for carbon based nanomaterials. While XRD established the core graphitic crystalline structure of the CQDs, FTIR spectroscopy was subsequently performed to identify the specific surface functional groups decorating this crystalline framework. Furthermore, FTIR was extended to the composite hydrogel system to demonstrate how these surface groups participate in intermolecular crosslinking to form the 3D network.

Fig. 1(b) shows the FTIR pattern of CQD. The CQD exhibits absorption bands at 3346 cm cm^−1^, 2138 cm cm^−1^, 1639 cm cm^−1^, 1579 cm cm^−1^, 902 cm cm^−1^, 812 cm cm^−1^, 554 cm cm^−1^, and 612 cm cm^−1^. The peak at 3346 cm^−1^ represents the stretching vibration of the hydroxyl group (O–H). The absorbance peaks at 554 cm^−1^ and 1639 cm^−1^ indicated the presence of secondary amide C–O and C=O groups. The peaks at 2138 cm^−1^ and 1279 cm cm^−1^ are associated with C–N bonds (amine and aromatic amine). Peaks at 902 cm^−1^, 812 cm^−1^, and 612 cm^−1^ corresponded to the bending of the aromatic C–H plane. The FT-IR analysis indicates the presence of multiple functional groups on the surface of CQDs, including carboxyl and amino groups.^40^ Having confirmed the surface functional states and passivating groups *via* FTIR, PL spectroscopy was conducted to evaluate how these surface states govern the optical and fluorescence properties of the CQDs.

The PL spectrum of the synthesized CQDs shows a prominent emission peak at approximately 2.875 eV (corresponding to ∼432 nm) is shown in Fig. 1(c). This strong blue emission under UV excitation is a characteristic feature of CQDs, arising from quantum confinement effects and surface defect states. The broad emission profile further indicates the presence of various emissive traps on the CQD's surface, which is typical for carbon based nanomaterials.^41^ These optical features confirm the formation of CQDs with size dependent luminescence properties suitable for bioimaging and sensing applications. To correlate these surface-state driven photoluminescent properties and crystal phase parameters with exact physical dimensions and morphology, TEM is used.

### 3.2. TEM

**Fig. 2:**
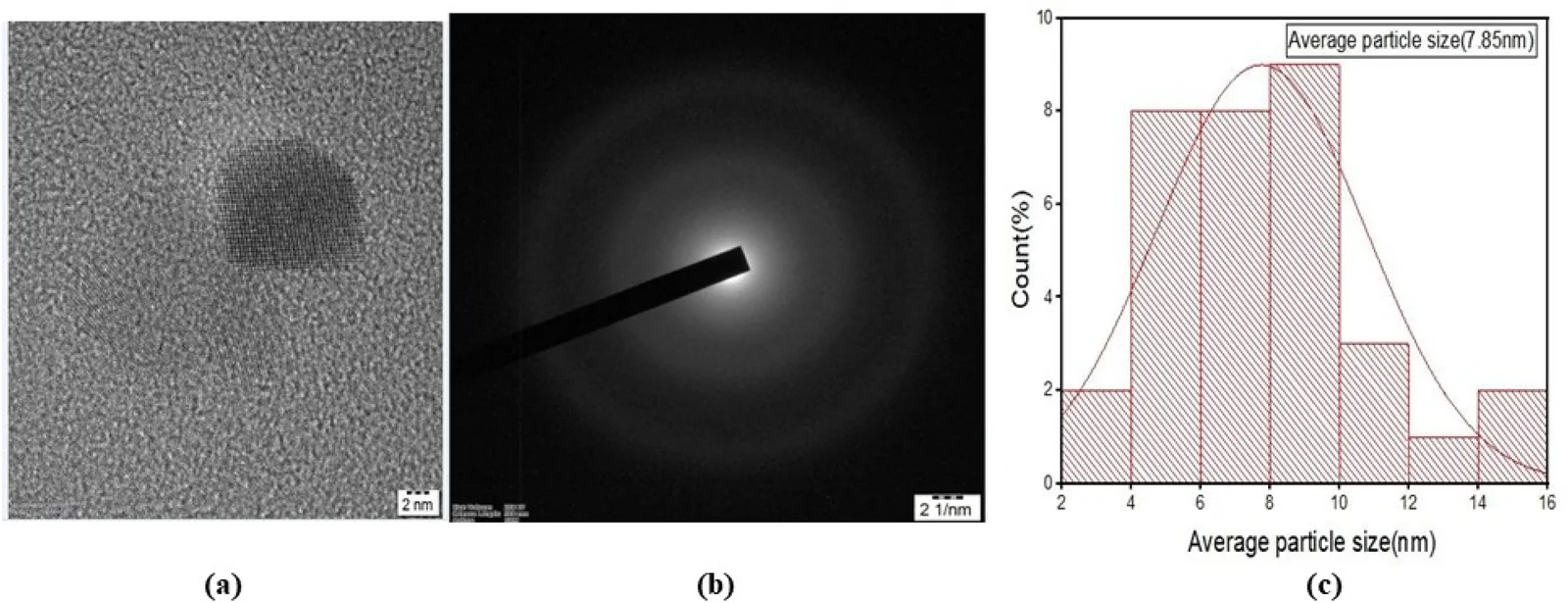
Morphological and structural characterization of CQD: (a) TEM image showing the morphology and nanoscale size of the CQDs, (b) SAED pattern indicating their structural characteristics, and (c) particle size distribution histogram obtained from the TEM images.

The TEM image of CQDs in Fig. 2(a) show that they are primarily spherical in morphology.

The SAED pattern of CQD is given in Fig. 2(b). The SAED image indicate that CQDs is amorphous in nature.^42^ The image displays broad, diffuse concentric rings rather than sharp, discrete spots or well defined rings. This is a hallmark of an amorphous structure, indicating lack of long-range crystallinity. The rings correspond to short-range order within the material, often present in carbon based amorphous materials due to random stacking or graphitic domains. The histogram generated from the statistical analysis of around 40 particles using Image software, is shown in Fig. 2(c). The Gaussian fitting curve shows the particle size distribution of the CQDs, ranging from 2 to 16 nm, with an average diameter of approximately 7.85 nm. To verify that the spherical nanostructures observed under TEM consisted of the targeted carbonaceous framework, EDX was performed to analyze the elemental composition.

The elemental composition and purity of the synthesized CQDs were investigated using Energy Dispersive X-ray (EDX) spectroscopy. The quantitative results, summarized in Table 1, confirm the presence of carbon (C), nitrogen (N), and oxygen (O) as the primary constituent elements. Carbon exhibits the highest atomic concentration at 70.72%, affirming the carbonaceous core of the nanoparticles. The significant presence of oxygen (13.53%) and nitrogen (15.75%) indicates successful surface functionalization with oxygen- and nitrogen-containing groups. These heteroatoms are crucial for enhancing aqueous solubility and introducing surface defects that facilitate radiative recombination, thereby improving photoluminescence. The absence of any other significant peaks in the EDX spectrum (with a normalized sum of 100%) underscores the high purity of the synthesized nanomaterial, confirming that the green-mediated or chemical precursors were fully processed without leaving detectable metallic or inorganic impurities. Because the elemental surface moieties identified by EDX dictate the electrokinetic behavior in liquid dispersion, Zeta potential analysis was conducted to quantify surface charge and colloidal stability.

**Table 1:** Elemental composition of carbon quantum dot determined by EDS, including the detected elements and their corresponding weight and atomic percentages

Element	At. No.	Mass (%)	Mass norm. (%)	Atom (%)
Carbon	6	65.83	66.02	70.72
Nitrogen	7	17.10	17.15	15.75
Oxygen	8	16.78	16.78	13.53
	**SUM**	**99.71**	**100.00**	**100.00**

### 3.3. ZETA potential

The surface charge and physical stability of the synthesized CQDs were evaluated *via* Zeta potential analysis. The particles exhibited a negative Zeta potential of −7.53 mV. This specific value indicates a net negative surface charge, likely attributed to the presence of oxygen-rich functional groups, such as carboxyl (–COOH) or hydroxyl (–OH) moieties. This is confirmed by FTIR. While a magnitude of 7.53 mV suggests moderate electrostatic repulsion, it is sufficient to maintain functional colloidal dispersion. This surface is critical for ensuring the nanoparticles remain stable in aqueous media, preventing excessive aggregation and facilitating effective interface interactions during subsequent material characterization and performance assays. To complement the crystalline insights from XRD and quantify the structural disorder (*I*_D_/*I*_G_ ratio) associated with these charged surface functional groups, Raman spectroscopy was used.

### 3.4. Raman spectroscopy and UV analysis

**Fig. 3:**
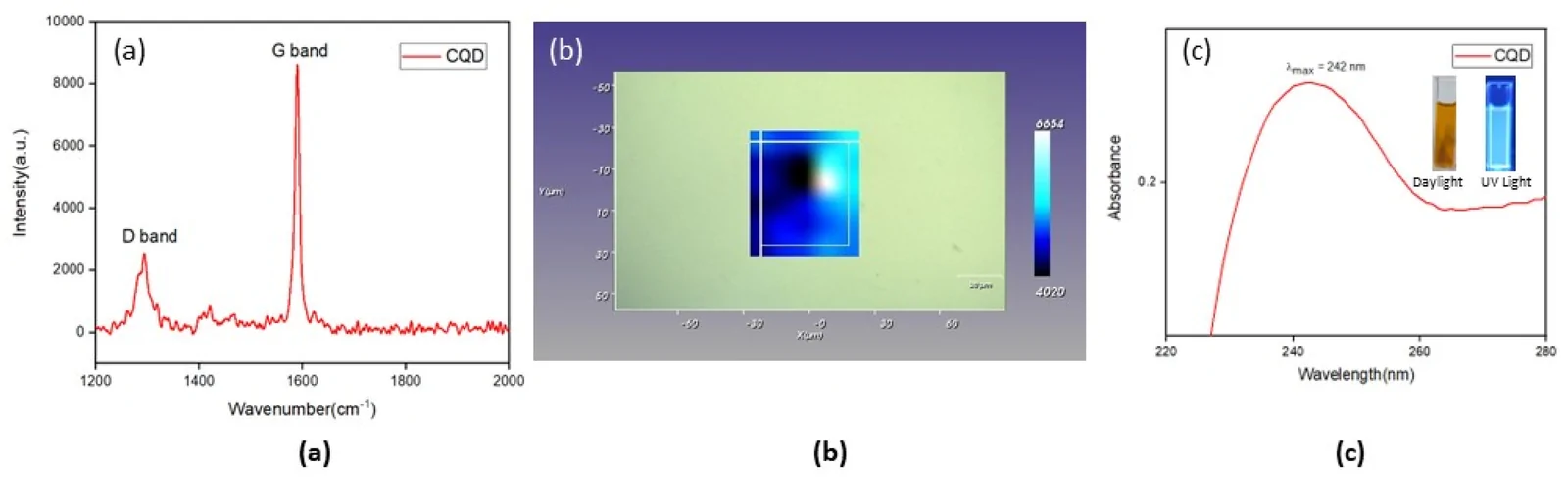
(a) Raman spectrum of CQD, (b) Raman mapping of CQD, (c) UV-Visible spectroscopy of CQD.

The Raman spectrum of the CQDs (Fig. 3(a)) displays two characteristic peaks: the D band at approximately 1294 cm^−1^ and the G band at 1590 cm^−1^. The D band originates from the A_1g_ breathing mode of disordered sp^2^ carbon atoms, while the G band corresponds to the E_2g_ in-plane stretching of crystalline graphitic carbon. Notably, the G band exhibits significantly higher intensity than the D band, resulting in a low *I*_D_/*I*_G_ ratio of approximately 0.28. This indicates a well-ordered sp^2^ carbon framework, confirming the successful formation of a graphitic core with minimal structural defects.

The Raman intensity mapping of the CQDs in Fig. 3(b), centred at the G-band frequency of 1618 cm^−1^, reveals a spatially heterogeneous distribution of sp^2^-hybridized graphitic domains across the sampled 150 × 100 µm area. The peak intensity of white/bright cyan regions, approximately 6654 counts) is localized in a central cluster approximately 20 µm in diameter, indicating a high density of crystalline CQDs or localized aggregation. Conversely, the transition to lower intensity values (dark blue/black regions, approximately 4020 counts) suggests areas of lower concentration or increased structural disorder. The observed blue shift of the G-band to 1618 cm^−1^, compared to the typical 1580 cm^−1^ seen in bulk graphite, is attributed to the quantum confinement effects and surface functionalization characteristic of nanoscale carbon structures. Bringing together the structural parameters from XRD/Raman and the functional surface states from FTIR/EDX, UV-Visible absorption spectroscopy was performed to correlate the electronic band transitions with the established structure.

Fig. 3(c) shows the UV-Visible absorption spectrum of the synthesized CQDs reveals a prominent characteristic peak that confirms their successful formation and structural properties. As illustrated in the graph, the CQDs exhibit a distinct absorption maximum (*λ*_max_) at 242 nm. This sharp peak in the deep ultraviolet region is primarily attributed to the π–π* electronic transitions of the C=C bonds within the sp^2^-hybridized carbon framework of the quantum dot core.

The well-defined nature of the peak suggests a consistent electronic environment and high structural integrity of the aromatic domains. Furthermore, the absorption profile shows a gradual tailing into the longer wavelength regions, a common feature in CQDs typically associated with π–π* transitions of surface functional groups (such as C=O or C–OH moieties). These results indicate that the CQDs possess a carbonaceous core with significant surface passivation, making them electronically active and suitable for subsequent biological and chemical applications. The synthesized CQDs displayed a light brown hue under ambient lighting. Upon excitation at 365 nm *via* UV irradiation, the solution exhibited intense blue-green PL as shown in Fig. 3(c).

### 3.5. Fluorescence quantum yield

The synthesized CQDs exhibit strong blue fluorescence with an emission peak at 432 nm, and the fluorescence quantum yield was calculated to be ∼31% using quinine sulfate as the reference standard.

### 3.6. Minimum inhibitory concentration

The antibacterial efficacy of the synthesized CQDs was evaluated against four clinically significant wound isolates: *Escherichia coli, Enterococcus faecalis*, *Staphylococcus aureus*, and *Pseudomonas aeruginosa.* Initial evaluations at a sub-inhibitory concentration of 10 mg mL^−1^ demonstrated partial, concentration-dependent bacteriostatic activity, yielding growth inhibition values of 47.63% for *E. coli*, 68.4% for *P. aeruginosa*, 22.7% for *S. aureus*, and 41.59% for *E. faecalis*. Upon evaluating the stepwise concentration gradient (10, 12, 14 and 16 mg mL^−1^), progressive growth suppression was observed across all tested strains. Complete bacterial growth inhibition (90–100%) was achieved at 16 mg mL^−1^, which is established as the true MIC for the synthesized CQDs. The observed sensitivity trends indicate that Gram-negative strains (*P. aeruginosa* and *E. coli*) exhibit higher susceptibility to CQD exposure at lower doses compared to Gram-positive strains, attributed to distinct cell wall architecture and electrostatic membrane interactions.

### 3.7. Well diffusion

**Fig. 4:**
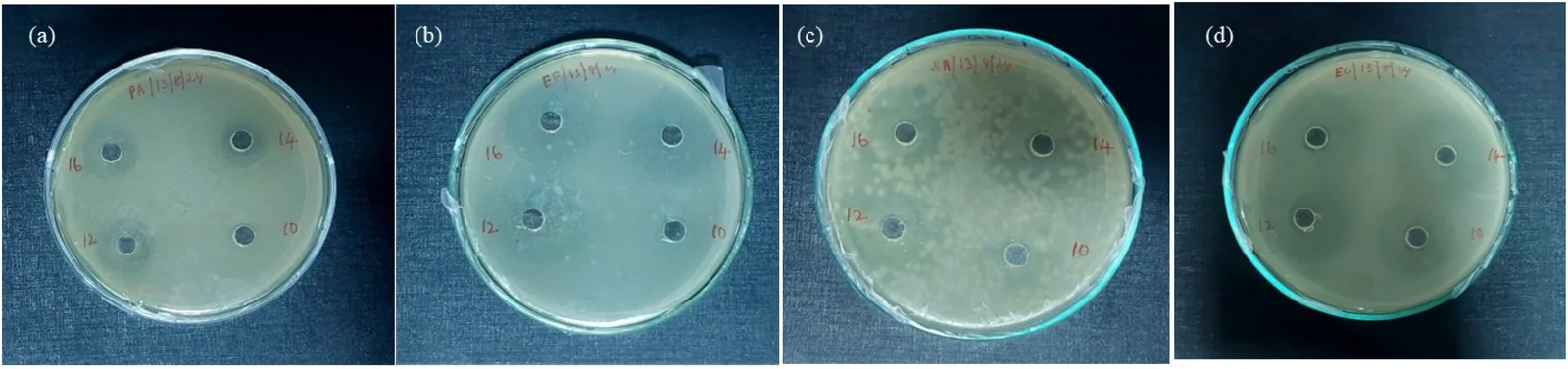
Well diffusion of CQD for (a) *P. aeruginosa*, (b) *E. faecalis*, (c) *S. aureus* and (d) *E. coli*.

Fig. 4 shows the zone of inhibition of CQD against wound pathogens at four concentrations (10, 12, 14, and 16 mg mL^−1^). Tested against *P. aeruginosa*, *E. faecalis*, *S. aureus*, and *E. coli*, the assessment reveals a clear concentration dependent increase in inhibition zones for all bacteria. To ensure precision, inhibition zone diameters were measured across the outer edge of complete macroscopic bacterial inhibition using digital Vernier calipers to calculate the mean diameter, adhering to standard CLSI guidelines where minor peripheral colonies are excluded. Among Gram-negative strains, *P. aeruginosa* exhibited lower sensitivity (1 mm to 8 mm), while *E. coli* showed slightly higher inhibition (2 mm to 8 mm). In contrast, Gram-positive bacteria demonstrated stronger responses. *E. faecalis* inhibition zones increased from 1 mm to 10 mm, and *S. aureus* exhibited the highest overall sensitivity, ranging from 5 mm to 9 mm.

The greater inhibition observed in Gram-positive bacteria is attributed to their simpler, single layered peptidoglycan cell wall, which allows easier penetration of CQD. Conversely, Gram-negative bacteria possess an outer lipid membrane that restricts the diffusion of antimicrobial agents. The results confirm that CQD is effective against the tested bacteria, and its activity strengthens progressively with increasing concentration.^43^ The specific diameters are provided in Table 2.

**Table 2:** Zone of inhibition values for CQD

BACTERIA/CONC	10 mg mL^−1^	12 mg mL^−1^	14 mg mL^−1^	16 mg mL^−1^
*Pseudomonas aeruginosa*	1.0 ± 0.1 mm	4.0 ± 0.2 mm	6.0 ± 0.3 mm	8.0 ± 0.2 mm
*Enterococcus faecalis*	1.0 ± 0.2 mm	4.0 ± 0.3 mm	6.0 ± 0.2 mm	10.0 ± 0.3 mm
*Staphylococcus aureus*	5.0 ± 0.3 mm	7.0 ± 0.4 mm	8.0 ± 0.3 mm	9.0 ± 0.4 mm
*Escherichia coli*	2.0 ± 0.2 mm	5.0 ± 0.3 mm	6.0 ± 0.4 mm	8.0 ± 0.3 mm

### 3.8. Growth curve

**Fig. 5:**
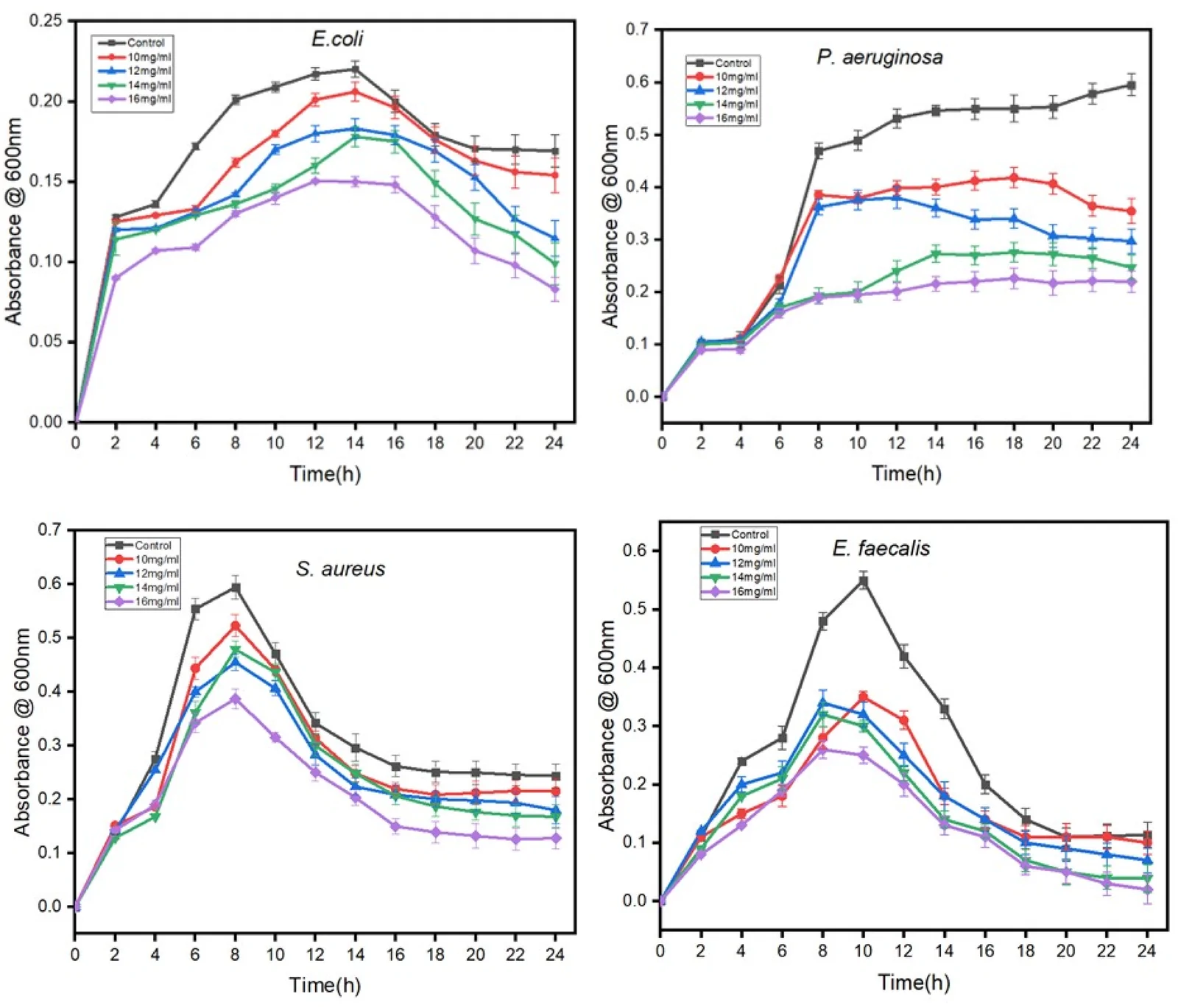
Growth curve of CQD treated bacteria.

Fig. 5 shows the growth kinetics of *E. coli, P. aeruginosa*, *S. aureus*, and *E. faecalis* treated with CQDs (10, 12, 14, and 16 mg mL^−1^). All species exhibit a lag phase during the initial 0–2 h, though *S. aureus* shows a slightly extended period of 3 h. Control cultures enter the exponential phase with sharp absorbance rises between 8–12 h.

At the 24th h, inhibition profiles reveal a strong dose dependent effect. *E. coli* shows inhibition increasing from 15% to 50% across the concentration range. *P. aeruginosa* exhibits a stronger response, ranging from 40% to 60%. Among Gram-positive strains, *S. aureus* shows moderate inhibition (20% to 45%), while *E. faecalis* displays the highest susceptibility, reaching 80% inhibition at 16 mg mL^−1^.

For the growth kinetics study, post-hoc analysis revealed that CQD-treated groups (12, 14, and 16 mg mL^−1^) showed statistically significant reductions in bacterial growth compared to the control (*p* < 0.05), with the 16 mg mL^−1^ group demonstrating the most pronounced effect across all strains. Significant differences were also observed between lower (10 mg mL^−1^) and higher concentrations (14 and 16 mg mL^−1^), confirming a dose-dependent antibacterial response. The stationary phase, typically 12–18 h in controls, is significantly shortened and shifted earlier in treated groups, reflecting disrupted metabolic equilibrium. While the death phase begins after 18 h in controls, CQD treated bacteria enter this phase much earlier. Notably, *S. aureus* and *E. faecalis* show the most rapid transition into the decline phase. Overall, CQDs exert a concentration dependent effect by slowing exponential growth, reducing stationary stability, and inducing premature cell death across all tested bacteria.^44^ While the well diffusion method provided preliminary evidence of antibacterial activity, the growth kinetics study offers a more robust and quantitative evaluation of antibacterial potency. In contrast to standard antibiotics such as Ciprofloxacin, which typically exhibit rapid bactericidal action and near-complete suppression of bacterial growth within a short duration, the CQDs in this study demonstrate a concentration-dependent modulation of bacterial growth dynamics. Specifically, CQD treatment results in an extended lag phase (∼2–3 hours in most strains, with a prolonged lag in *S. aureus*), along with a reduction in overall growth of approximately 50–80% at 24 hours depending on the bacterial species. Furthermore, CQDs significantly alter the growth profile by shortening the exponential phase and inducing an earlier transition into the decline phase, indicating disruption of cellular metabolism and proliferation. This behavior suggests a sustained antibacterial effect rather than rapid bactericidal killing, likely mediated by progressive mechanisms such as ROS generation, membrane destabilization, and interference with quorum sensing pathways.

### 3.9. Colony count

**Fig. 6:**
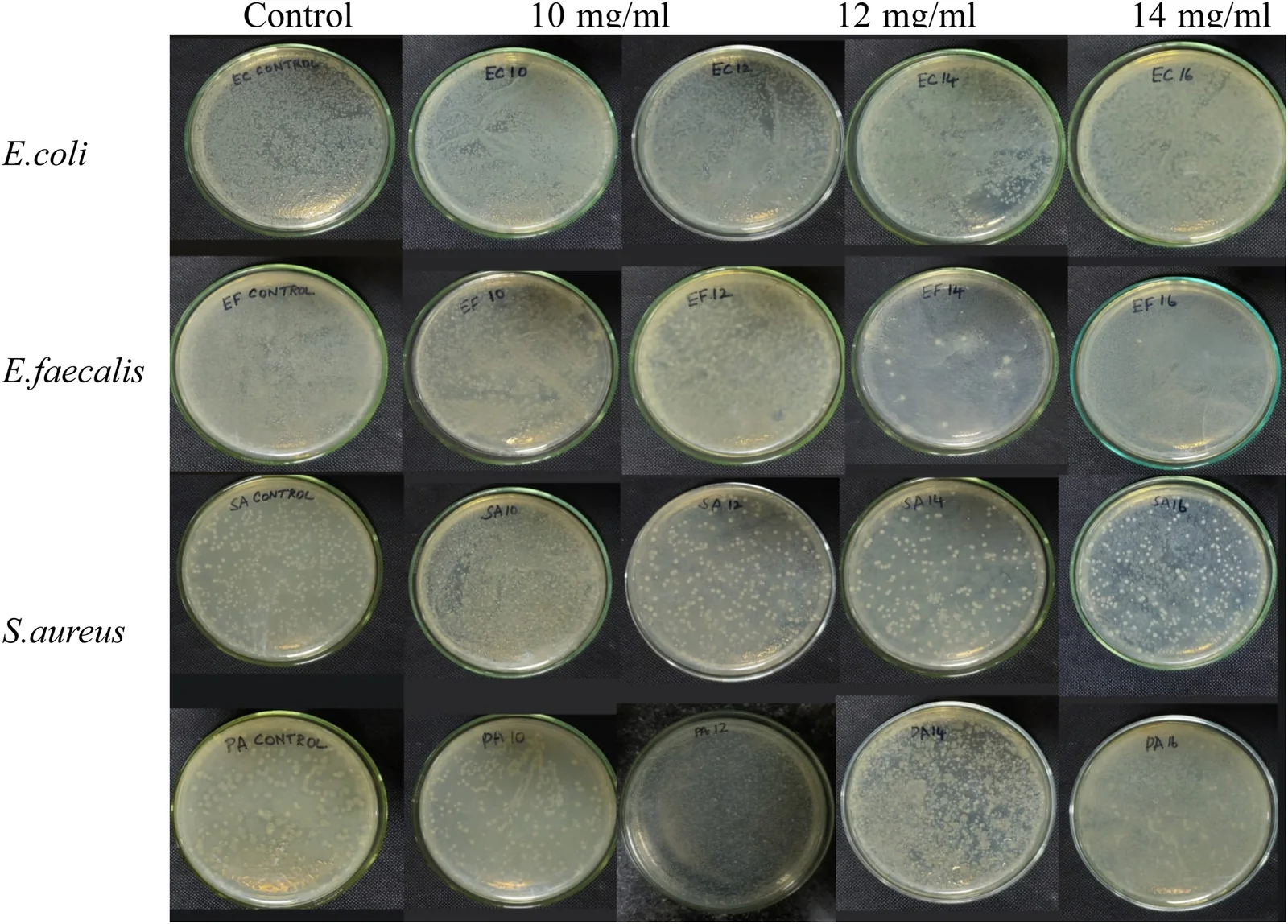
Colony count of CQD treated bacteria.

Bacterial viability is defined as the ratio of viable cells to the total bacterial population, including both live and dead cells. The viable count represents the number of live microorganisms present, which can be estimated from the colony count. In this study, two Gram-positive (*Staphylococcus aureus*, *Enterococcus faecalis*) and two Gram-negative (*Escherichia coli*, *Pseudomonas aeruginosa*) bacteria are analysed for their colony-forming ability. As shown in Fig. 6 and Table 3, the highest number of bacterial colonies are observed in the untreated control samples compared to those exposed to different concentrations of CQDs (10, 12, 14, 16 mg mL^−1^).

**Table 3:** Percentage bacterial growth inhibition (%) of four wound pathogens exposed to increasing concentrations (10–16 mg m^−1^) of CQDs

Pathogen	Evaluation parameter	Control	10 mg mL^−1^	12 mg mL^−1^	14 mg mL^−1^	16 mg mL^−1^
*E. coli*	Inhibition (%)	0.0%	31.3%	65.2%	88.4%	96.3
*E. faecalis*	Inhibition (%)	0.0%	32.3%	64.5%	85.2%	99.4
*S. aureus*	Inhibition (%)	0.0%	34.2%	52.5%	58.9%	65.2
*P. aeruginosa*	Inhibition (%)	0.0%	13.3%	41.4%	63.3%	77.3

Notably, at a concentration of 16 mg mL^−1^, *Enterococcus faecalis* exhibits the lowest colony count among all tested strains, achieving the highest growth inhibition of 99.4%, followed by *Escherichia coli* (96.3%), *Pseudomonas aeruginosa* (77.3%), and *Staphylococcus aureus* (65.2%). At lower treatment doses (10–14 mg mL^−1^), concentration-dependent inhibition was already evident, with initial growth suppression at 10 mg mL^−1^ recording 31.3% (*E. coli*), 32.3% (*E. faecalis*), 34.2% (*S. aureus*), and 13.3% (*P. aeruginosa*).

The results clearly indicate that colony formation decreases systematically with increasing CQD concentration. This decline is attributed to the interaction between CQDs and bacterial cells, where CQDs disrupt the bacterial cell membrane and intracellular components. The reduction in colony numbers directly reflects decreased bacterial viability at higher CQD concentrations. CQDs compromise cellular integrity by inducing membrane damage, oxidative stress, and leakage of intracellular constituents, thereby hindering essential metabolic processes. This disruption ultimately inhibits bacterial growth and division, resulting in fewer viable cells capable of forming visible colonies.^45^

### 3.10. Biofilm disruption

Biofilms are structured microbial communities embedded within a self-produced extracellular polymeric substance (EPS) matrix. This matrix restricts antimicrobial penetration and facilitates quorum sensing, granting enhanced bacteria resistance to host immune defenses and antibiotics.^43^ In chronic wounds, pathogens like *S. aureus*, *E. faecalis*, *P. aeruginosa,* and *E. coli* form biofilms that delay healing and serve as reservoirs for recurrent infection.

**Fig. 7:**
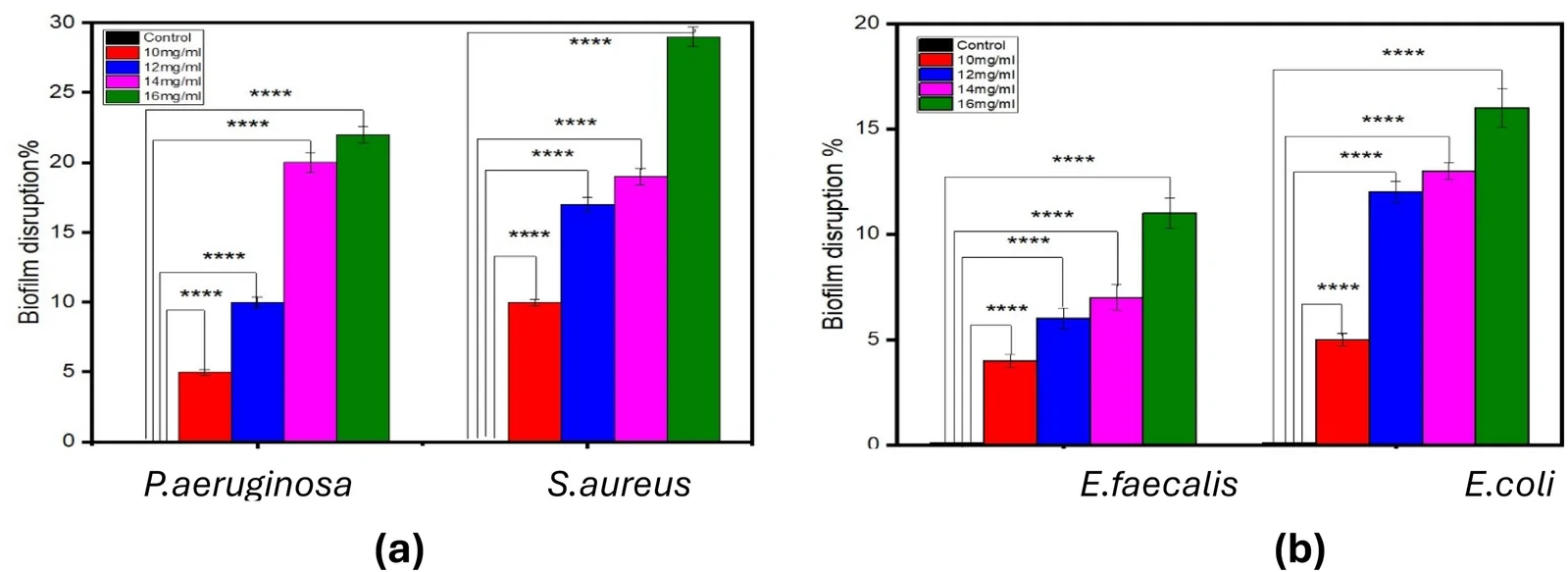
Biofilm disruption by CQD, (a) *E. faecalis and S. aureus* (b) *E. coli and P. aeruginosa*. **** represents a significant difference between control and different concentrations (*p* value < 0.0001).

Fig. 7 illustrates the dose dependent efficacy of CQD in disrupting preformed biofilms at 10, 12, 14, and 16 mg mL^−1^. Across all species, disruption increases progressively with concentration. Among the tested pathogens, *S. aureus* exhibits the highest susceptibility, with disruption reaching approximately 29% at 16 mg mL^−1^ (*p* < 0.0001). This response is linked to the thick peptidoglycan layer of Gram-positive cell walls.

In contrast, *P. aeruginosa* demonstrates a peak disruption of 22% at 16 mg mL^−1^ (*p* < 0.0001). For *E. faecalis* and *E. coli*, disruption is moderate, reaching approximately 11% and 16%, respectively. These findings confirm that CQDs possess potent, dose-dependent antibiofilm activity against both Gram-positive and Gram-negative bacteria, serving as a promising strategy for managing biofilm associated chronic wound infections.

### 3.11. Biofilm formations

**Fig. 8:**
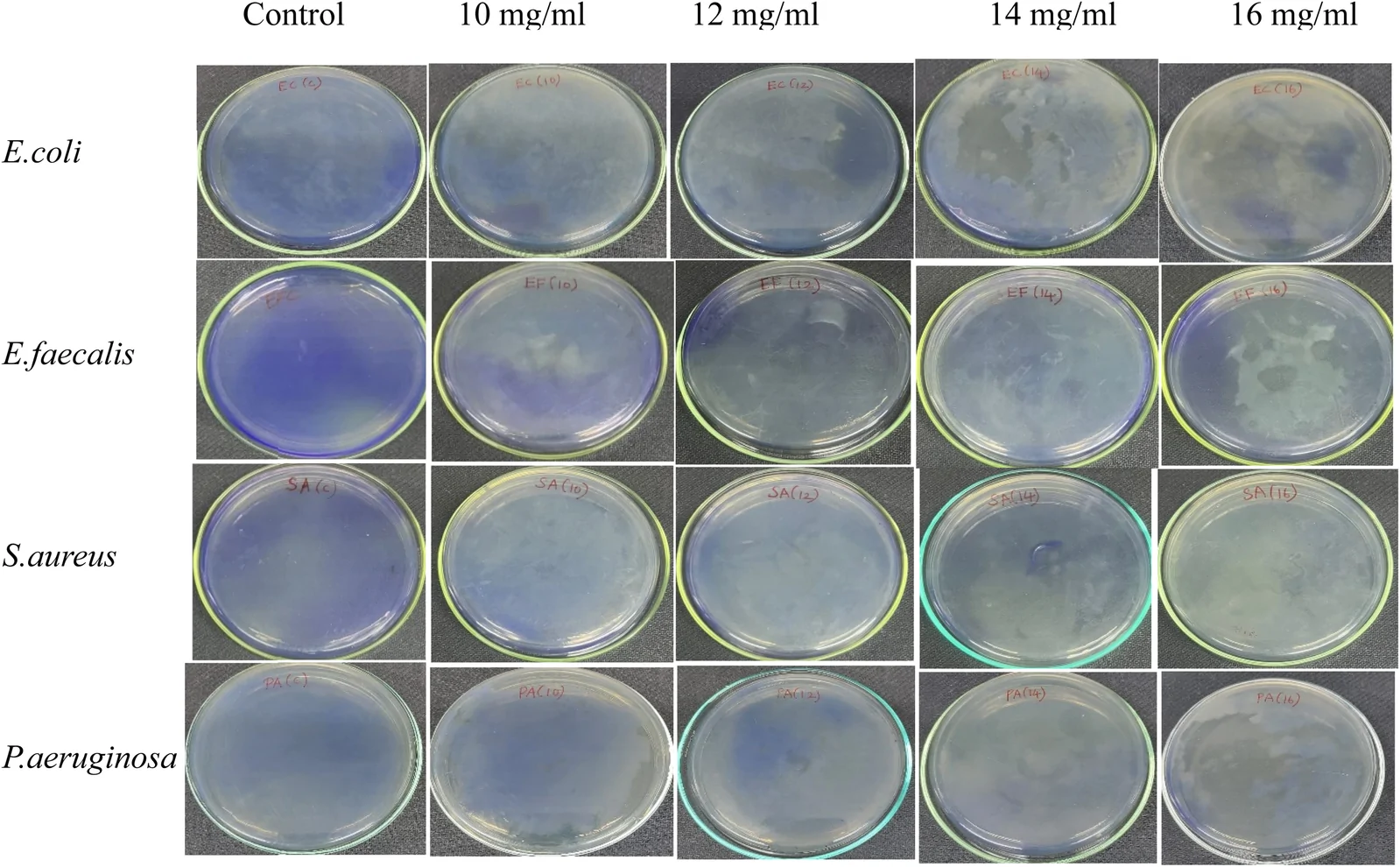
Biofilm formation assay of the synthesized material against the tested bacterial strains, showing the inhibitory effect of the treatment on bacterial biofilm formation compared with the untreated control.

Biofilm formation is a critical virulence strategy where bacteria reside within a self-produced EPS matrix to evade host immunity and resist antibiotics.^43^ This study evaluates CQD anti biofilm activity against *E. coli*,*E. faecalis*, *S. aureus*, and *P. aeruginosa* using crystal violet staining at concentrations of 10, 12, 14, and 16 mg mL^−1^. The results in Fig. 8 show a clear, concentration dependent inhibition across all strains. In Gram negative bacteria, *E. coli* shows near complete inhibition at 16 mg mL^−1^, potentially through sensing interference, while *P. aeruginosa* exhibits substantial biomass reduction even at 10 mg mL^−1^. Among Gram-positive strains, *E. faecalis* and *S. aureus* demonstrate stepwise decreases in staining, indicating reduced EPS production and direct cell wall interaction. Sensitivity variations reflect structural differences. Gram-negative strains show abrupt reductions at high doses, whereas Gram-positive strains respond more gradually. Overall, CQDs demonstrate broad spectrum anti biofilm potency, effectively preventing development across diverse bacterial types and highlighting their potential for treating biofilm associated infections.

### 3.12. Reactive oxygen species

**Fig. 9:**
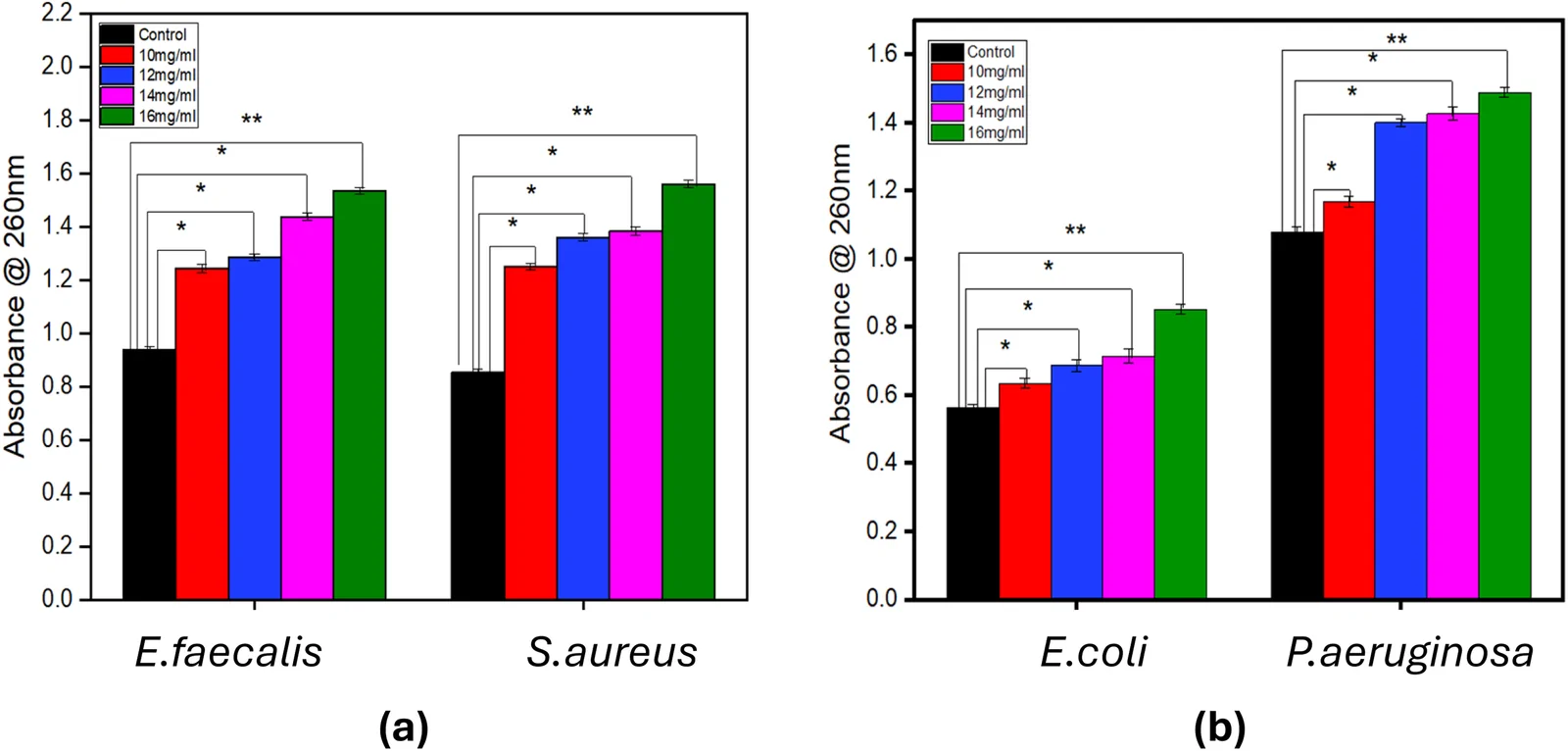
ROS production by CQD (a) *E. faecalis* and *S. aureus* (b) *E. coli* and *P. aeruginosa*. * represents a significant difference between control and different concentrations (*p* value < 0.05).

**Fig. 10:**
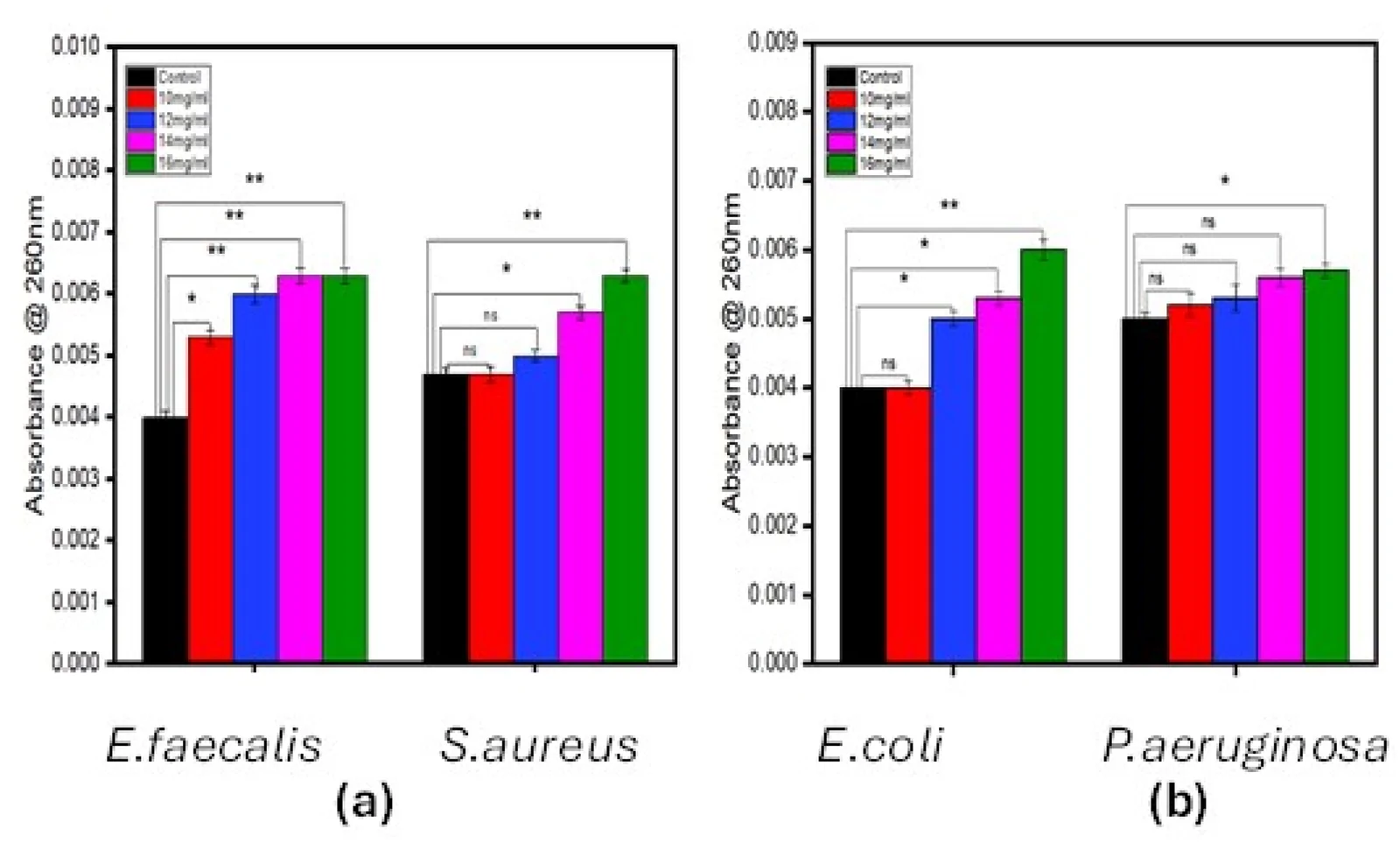
Cell membrane integrity by CQD (a) *E. faecalis* and *S. aureus* (b) *E. coli* and *P. aeruginosa*. * represents a significant difference between control and different concentrations (*p* value < 0.05) and ns. represents not significant.

Fig. 9 shows the intracellular production of ROS in response to CQDs was quantified for *E. faecalis*, *S. aureus*, *E. coli*, and *P. aeruginosa*. As illustrated in Fig. 10, the treatment induced a clear, dose-dependent elevation in ROS levels across all bacterial strains when compared to their respective untreated controls. A comparative analysis reveals that the Gram-positive strains*, E. faecalis* and *S. aureus*, exhibited a robust antioxidant response, with significant increases in ROS accumulation starting from the lowest concentration of 10 mg mL^−1^ (*p < 0.05). Among the Gram-negative bacteria, *P. aeruginosa* showed the highest susceptibility to oxidative stress, reaching extreme statistical significance (**p < 0.01) at the 16 mg mL^−1^ dosage. While *E. coli* also displayed an upward trend, its ROS levels remained lower in absolute magnitude compared to the other strains, though still maintaining statistical significance across the concentration gradient. These findings indicate that the antimicrobial efficacy of CQDs is heavily predicated on the induction of oxidative stress. The surge in intracellular ROS likely leads to downstream lipid peroxidation and protein carbonylation, ultimately resulting in cellular death.

### 3.13. Cell membrane integrity

The impact of CQDs on the cell membrane integrity of four bacterial strains—*E. faecalis*, *S. aureus*, *E. coli*, and *P. aeruginosa* was evaluated by measuring the leakage of intracellular constituents at 260 nm. As shown in Fig. 10, all treated groups exhibit a dose-dependent increase in absorbance relative to the untreated control, signifying varying degrees of membrane compromise. Among the Gram-positive strains, *E. faecalis* demonstrated the highest susceptibility, with statistically significant leakage (*p* < 0.05) observed across all concentrations (10–16 mg mL^−1^). *S. aureus* showed a similar upward trend, achieving significant membrane disruption (**p < 0.01) at the maximum concentration of 16 mg mL^−1^. In contrast, the Gram-negative strains, *E. coli* and *P. aeruginosa*, exhibited more robust membrane stability. While higher concentrations (14 and 16 mL) induced detectable leakage (*p* < 0.05), the lower doses (10 mg mL^−1^) remained non-significant (ns). These results suggest that CQD-induced antimicrobial activity is mediated by the mechanical disruption of the lipid bilayer, leading to the irreversible release of cytoplasmic materials.

### 3.14. Exopolysaccharide

**Fig. 11:**
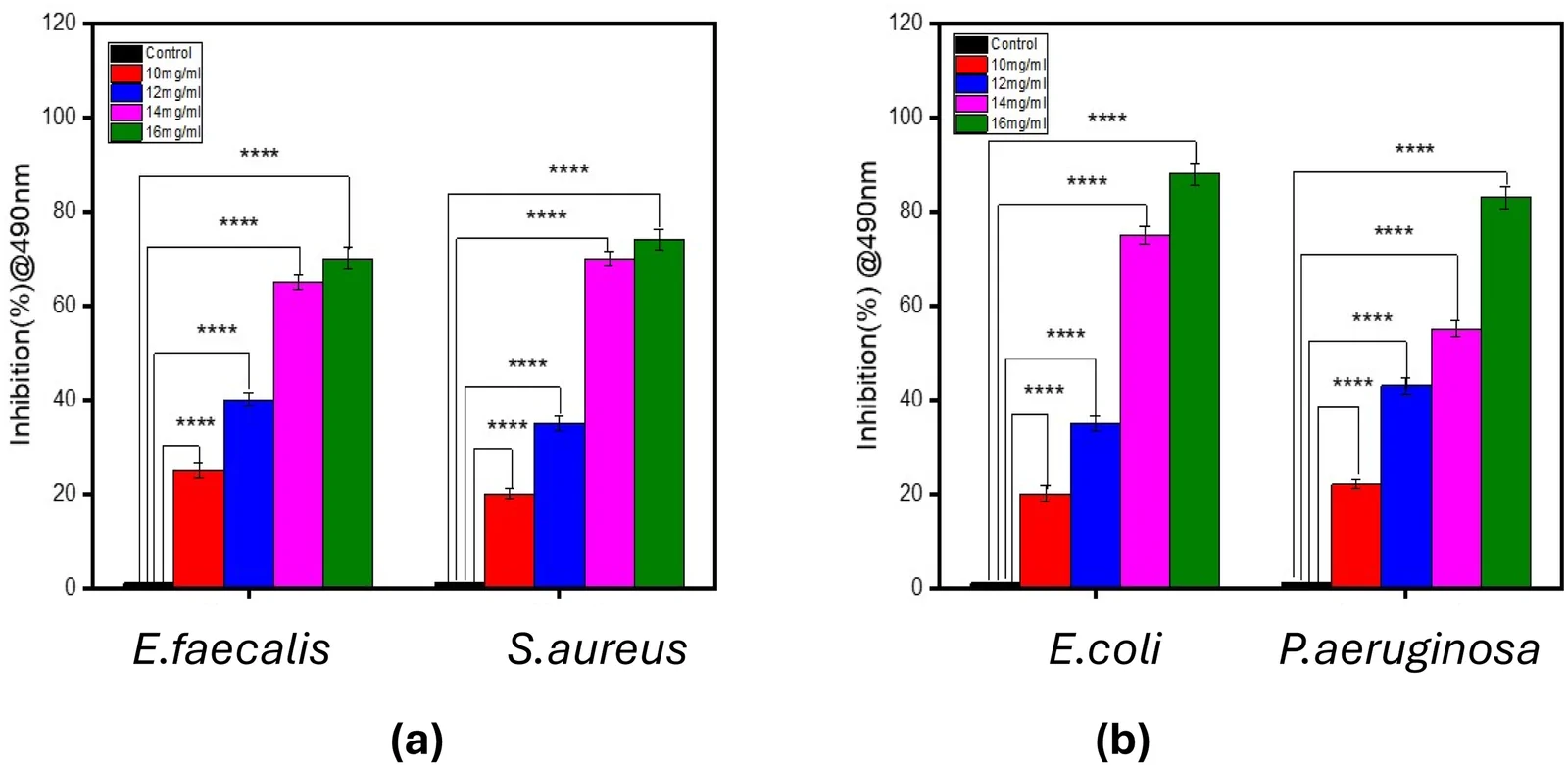
Exopolysaccharide inhibition by CQD, (a) *E. faecalis* and *S. aureus* (b) *E. coli* and *P. aeruginosa*. **** represents a significant difference between control and different concentrations (*p* value < 0.0001).

The inhibitory effect of CQDs on exopolysaccharide (EPS) production across four bacterial strains is shown in Fig. 11. All strains exhibit a clear dose dependent inhibition pattern (*p* < 0.0001). For Gram-positive bacteria, *S. aureus* inhibition increases from ∼24% at 10 mg mL^−1^ to ∼75% at 16 mg mL^−1^, while *E. faecalis* shows a moderate range of ∼26% to ∼69%. Among Gram-negative strains, *P. aeruginosa* achieves approximately 82% inhibition at 16 mg mL^−1^. *E. coli* demonstrates the highest maximum inhibition at 89%, though it suggests a threshold dependent response due to lower efficacy at smaller doses.

These results suggest CQDs disrupt the polysaccharide matrix's structural integrity or induce degradative enzymes. In *P. aeruginosa*, the thin peptidoglycan layer may enhance CQD penetration and EPS degradation. Overall, CQDs show significant potential for antimicrobial strategies targeting EPS mediated biofilm formation.

### 3.15. Protein leakage

**Fig. 12:**
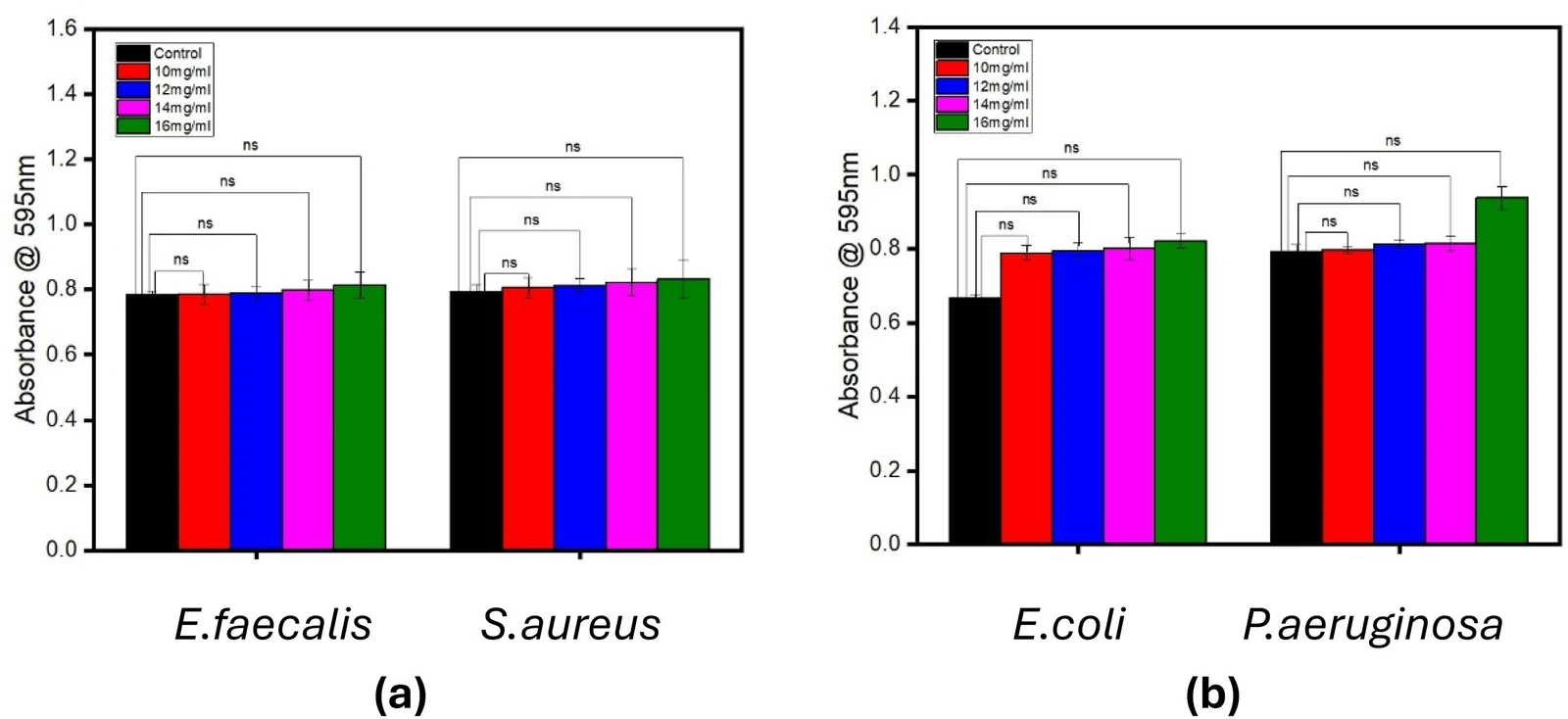
Protein leakage by CQD, (a) *E. faecalis* and *S. aureus* (b) *E. coli* and *P. aeruginosa*.

Protein leakage is a hallmark of bacterial membrane disruption, occurring when intracellular proteins diffuse into the extracellular medium due to loss of envelope integrity. In this study, protein leakage is measured in *P. aeruginosa*, *S. aureus*, *E. coli*, *E. faecalis* after treatment with CQDs (10–16 mg mL^−1^). As shown in Fig. 12, increased absorbance at 595 nm indicates protein release across all strains compared to controls. Although statistical analysis categorizes these differences as not significant (ns), a concentration dependent trend is evident, particularly in *E. coli* and *E. faecalis*. The modest extent of leakage suggests that these CQD concentrations partially disrupt the membrane without causing extensive rupture. Overall, the findings confirm that CQDs possess antimicrobial potential by targeting the bacterial cell envelope and inducing gradual protein efflux.

### 3.16. Lipid peroxidation

**Fig. 13:**
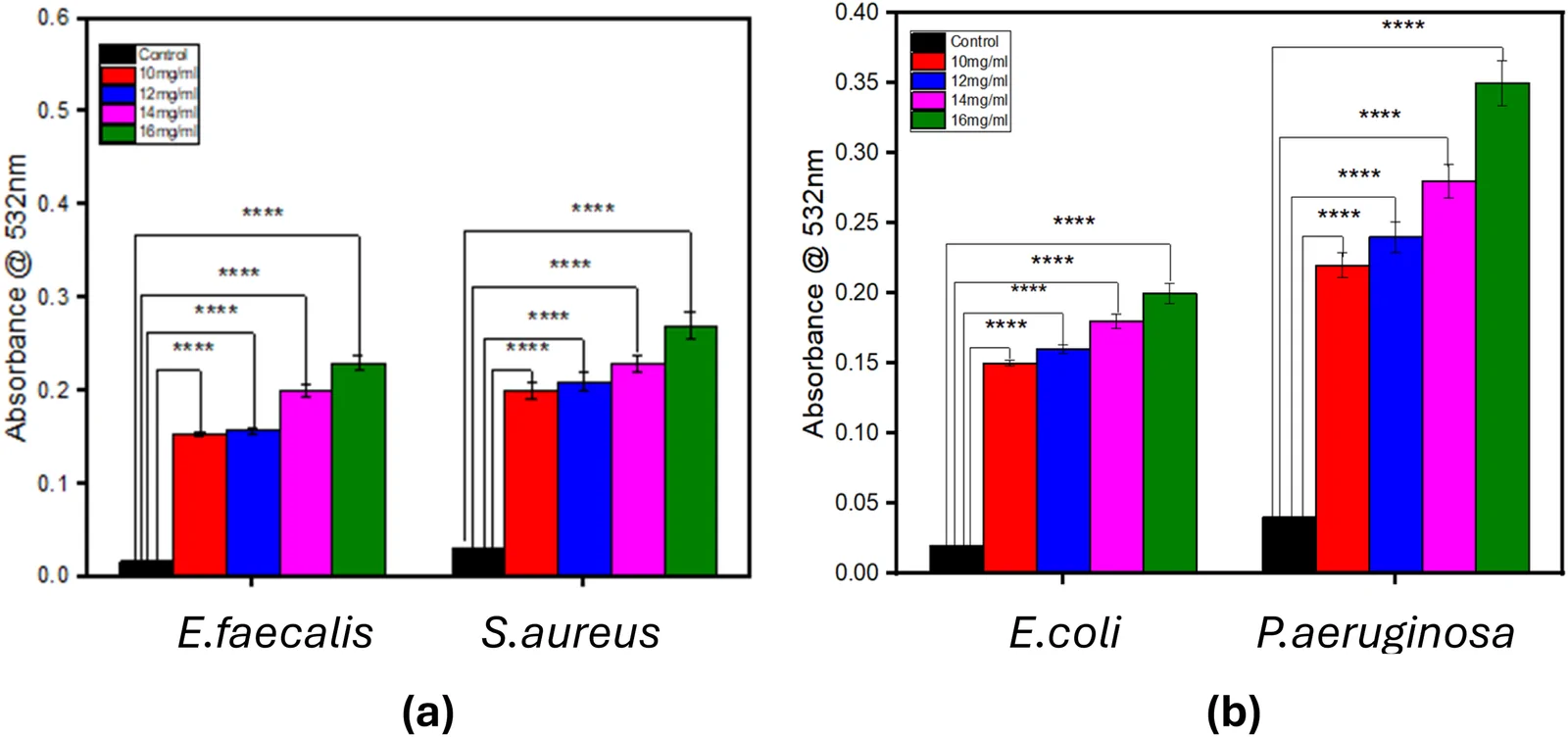
Lipid peroxidation by CQD (a) *E. faecalis* and *S. aureus* (b) *E. coli* and *P. aeruginosa*. **** represents a significant difference between control and different concentrations (*p* value < 0.0001).

Lipid peroxidation involves the oxidative degradation of polyunsaturated fatty acids in cell membranes, often initiated by ROS.^43^Fig. 13 displays a concentration dependent increase in lipid peroxidation across all strains, with results being statistically significant (*p* < 0.0001).

*P. aeruginosa* exhibits the highest damage, reaching an absorbance of ∼0.43 at 16 mg mL^−1^, followed by *S. aureus* at ∼0.32. Both *E. coli* and *E. faecalis* show notable increases, with absorbance values rising to ∼0.21 and ∼0.22, respectively. These results indicate severe oxidative stress and membrane destabilization.

The physicochemical properties of CQDs nanoscale size and reactive functional groups promote this damage by interacting with the bacterial envelope. This disruption compromises membrane integrity, leading to increased permeability and cell death. The effect is most pronounced at 16 mg mL^−1^, confirming that CQD induced oxidative stress is dose-dependent and effective across both Gram-positive and Gram-negative classifications.

### 3.17. FTIR of hydrogel

**Fig. 14:**
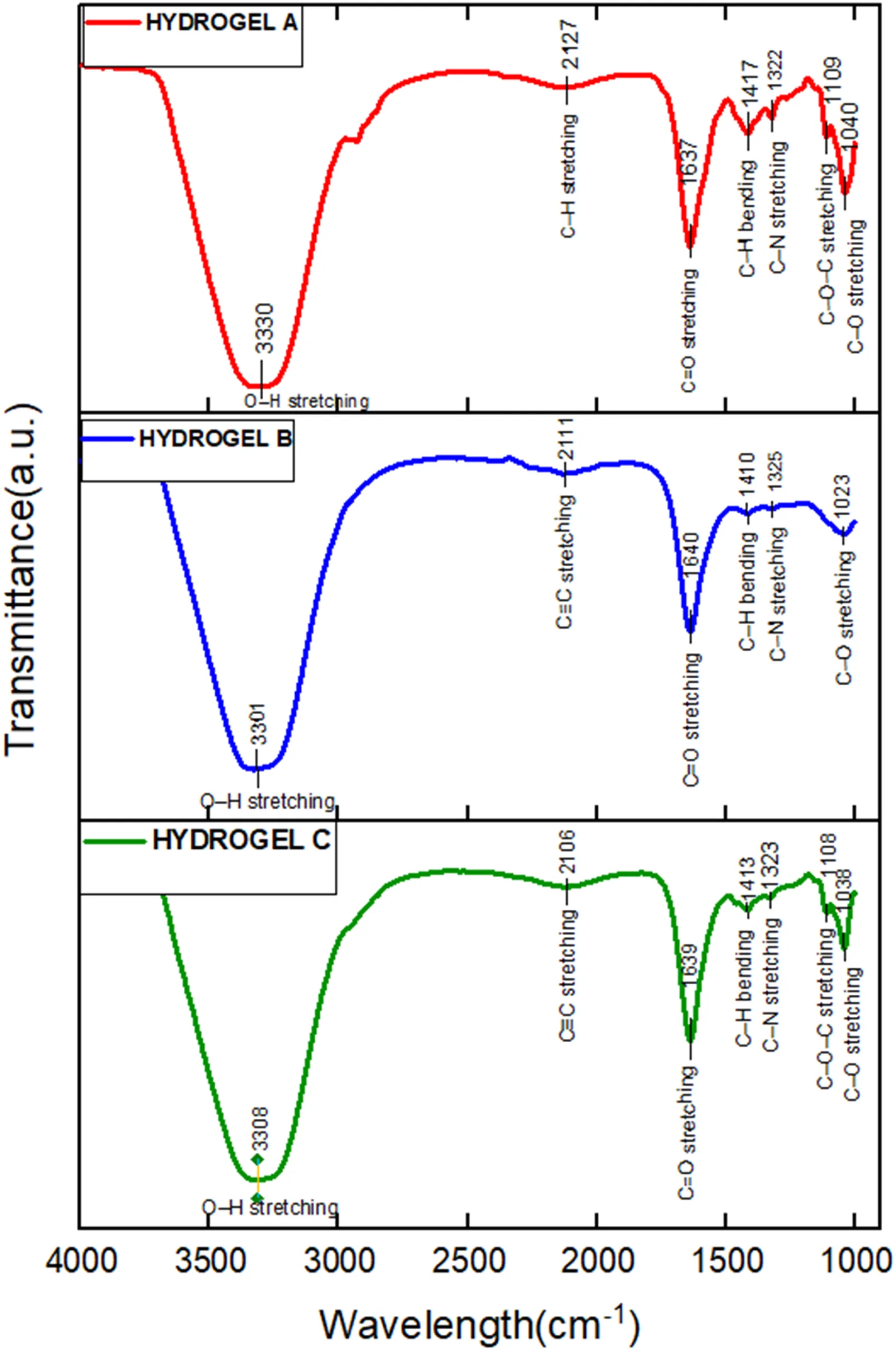
FTIR spectra of the prepared hydrogels showing the characteristic absorption bands associated with their functional groups and providing evidence of the chemical composition of the respective hydrogel formulations.

CQDs is incorporated into the hydrogel to improve its wound-healing efficacy by enhancing its biological activity and therapeutic performance and characterized to confirm the incorporation of CQD to hydrogel in FTIR. Fig. 14(a) shows the FTIR spectrum of hydrogel A (CMC, AV, glycerol). A broad band at 3330 cm^−1^ corresponds to O–H stretching, while a peak at 1637 cm^−1^ confirms C=O stretching from carboxyl groups. Glycosidic linkages and glycerol are identified by C–O–C and C–O stretching at 1109 and 1040 cm^−1^.

In hydrogel B containing CQDs (Fig. 14(b)), the O–H band shifts to 3301 cm^−1^, indicating hydrogen bonding between the hydrogel and CQD surface functionalities. Enhanced absorption at 1640 cm^−1^ (C=O) and a shift in C–O stretching to 1023 cm^−1^ further confirm successful CQD incorporation.

Hydrogel C loaded with quercetin and CQDs (Fig. 14(c)) exhibits an O–H band at 3308 cm^−1^, suggesting interactions with quercetin's phenolic groups. A strong peak at 1639 cm^−1^ links to quercetin's C=O group, while aromatic skeletal vibrations at 1413 cm^−1^ support quercetin integration. Bands at 1108 and 1038 cm^−1^ reflect both hydrogel and quercetin glycosidic structures.

FTIR analysis validates the successful fabrication of all hydrogels. The results confirm characteristic polysaccharide bands in the pure formulation, additional carbonyl/hydroxyl groups from CQDs in the second, and flavonoid-specific aromatic vibrations in the third, confirming quercetin incorporation. To verify the non-covalent interactions and physical crosslinking across the hydrogel network, comparative FTIR spectra of pure CQDs, hydrogel A, hydrogel B, and hydrogel C were analyzed (Fig. 14). The pure CQD nanoparticles in (Fig. 1(b)) displayed characteristic functional surface groups, including broad –OH stretching at 3380 cm^−1^ and C=O stretching at 1650 cm^−1^. For hydrogel A, the primary biopolymer backbone (CMC/AV) exhibited a prominent –OH absorption band at 3350 cm^−1^ and asymmetric COO^−^ stretching at 1595 cm^−1^. Upon integrating CQDs into the network (hydrogel B), the –OH band shifted significantly downward to 3310 cm^−1^ with increased peak broadening, indicating dense hydrogen-bonded physical crosslinking between the CQD surface groups and the biopolymer chains. In hydrogel C, the concurrent loading of quercetin caused a further redshift of the –OH vibration to 3275 cm^−1^ and a shift in the carboxylate COO^−^ peak to 1572 cm^−1^. These progressive spectral shifts confirm that CQDs and quercetin act as dynamic physical crosslinkers, forming a stable, non-covalently interacting supramolecular hydrogel matrix without compromising the fundamental polysaccharide backbone (evidenced by the preserved C–O–C stretching at 1032 cm^−1^. FTIR spectra confirmed the structural assembly of the hydrogel. Characteristic absorption peaks observed at 3300 cm^−1^ and 1650 cm^−1^ confirmed extensive intermolecular hydrogen bonding and successful crosslinking, providing structural rigidity to the polymeric matrix.

### 3.18. Rheology

**Fig. 15:**
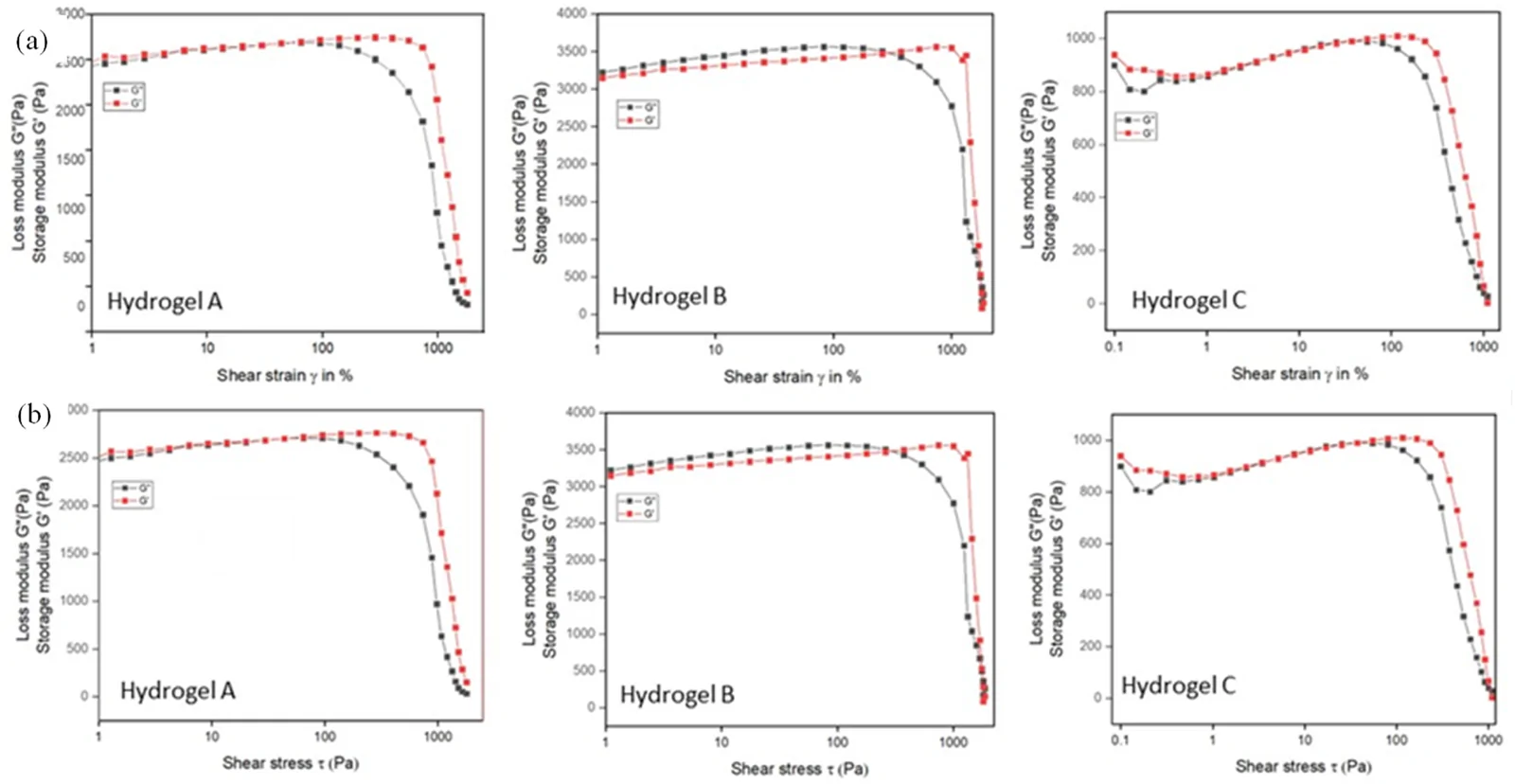
(a) Storage *G*′ and loss *G*″ moduli as a function of strain amplitude of hydrogel's A–C. (b) storage *G*′ and loss *G*″ moduli as a function of stress amplitude of hydrogel's A–C.

Quantitative shear stress–strain profiling and rheological mechanics and quantitative rheological evaluation *via* continuous shear stress (*τ*) *versus* shear strain (*γ*) profiling was conducted to substantiate the mechanical integrity, matrix stiffness, and yield dynamics of hydrogels A–C in Fig. 15(a) which shows the storage *G*′ and loss *G*″ moduli as a functions of strain amplitude and Fig. 15(b) shows the storage *G*′ and loss *G*″ moduli as a functions of stress amplitude of hydrogel's A–C. Hydrogel B exhibits the highest stiffness, whereas C is softest and A is intermediate. In each gel, storage modulus *G*′ is comparable to or slightly below loss modulus *G*″ in the linear viscoelastic regime (tan *δ* ≈ 1), indicating mixed elastic-viscous behavior. Plateau *G*′ and *G*″ values (from the linear regime) are ∼3.5 × 10^3^ and 3.8 × 10^3^ Pa for B, ∼2.3 × 10^3^ and 2.5 × 10^3^ Pa for A, and ∼0.85 × 10^3^ and 0.9 × 10^3^ Pa for C. These moduli persist up to 10^3^% strain (or ∼10^3^ Pa stress), beyond which *G*′ drops sharply, marking yield. B's high moduli make it likely to fail abruptly (brittle behavior), whereas C's low moduli imply greater compliance. Strain- and stress-sweep tests show similar plateau and yield transitions; only C exhibits a slight *G*′ dip at extremely low stress. Near-equivalence of *G*′ and *G*″ indicates significant energy dissipation in all gels. With testing frequency and temperature unspecified, these trends imply B is most mechanically robust (suitable for load bearing) and C is most dissipative (suitable for energy absorption). Furthermore, all formulations exhibited distinct yield stress (*τ*_*y*_) thresholds, demonstrating that the gel matrices remain dimensionally stable and solid-like under static resting conditions, yet yield smoothly into a fluid-like state once the applied shear exceeds the yield point. This quantitative shear response directly accounts for the observed macroscopic injectability, smooth spreadability, and structural retention required to conform over irregular wound topographies without matrix breakdown.

### 3.19. Injectability test for hydrogel

**Fig. 16:**
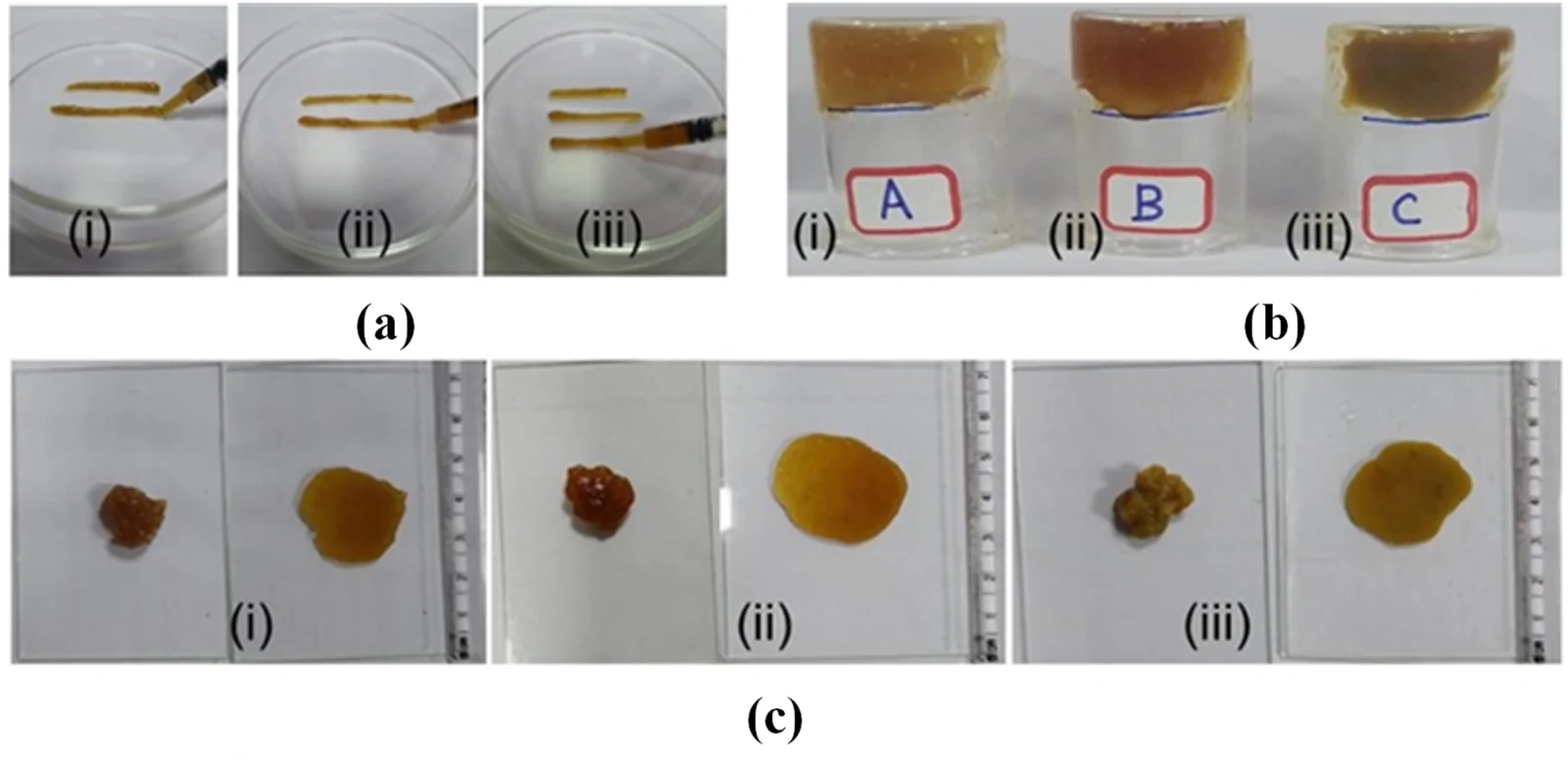
Evaluation of the physical and handling properties of the prepared hydrogels: (a) flow test, (b) inversion test, and (c) spreadability test for (i) pure hydrogel, (ii) CQD loaded hydrogel, and (iii) drug-loaded hydrogel formulations.

Fig. 16(a) demonstrates the successful extrusion of hydrogels A–C through a 21-gauge needle, validating their shear-thinning behaviour. This property temporarily lowers viscosity under pressure, facilitating effortless administration.^30^ All formulations extrude as smooth, continuous streams, ensuring uniform wound coverage without needle clogging. Notably, hydrogel C exhibits instantaneous structural recovery (thixotropy) upon exiting the needle, forming a stable viscoelastic matrix that prevents runoff. This smooth extrusion confirms that shear stress does not irreversibly damage the polymer network, preserving the hydrogels' structural integrity and therapeutic functionality. All formulations are fully injectable through 21 G needles, demonstrating rapid shear-recovery to maintain structural stability at the site of application. From an operational perspective, the formulation demonstrated superior injectability, extruding smoothly through a 21 G needle without clogging or phase separation under manual pressure.

### 3.20. Inversion test for hydrogel

Fig. 16(b) shows the inversion test of hydrogels A–C. After placing the vials containing the fully formed hydrogels in an inverted position for 72 h, no flow or deformation is observed in any of the three samples. The hydrogels remains firmly in place, demonstrating their robust internal structure and resistance to gravitational force. This successful outcome indicates that hydrogels A–C possess sufficient mechanical strength and self-supporting capability to maintain their intended shape and contact with the wound bed over an extended period, ensuring continuous therapeutic delivery and protection.^31^

### 3.21. Spreadability for hydrogel

Fig. 16(c) illustrates the spreadability of hydrogels A–C, which is a critical factor for achieving uniform wound coverage. Results show that hydrogel C exhibits the highest spreadability (4.50 g cm s^−1^), followed by hydrogel B (4.32 g cm s^−1^) and hydrogel A (4.16 g cm s^−1^).

This inverse correlation with viscosity indicates that hydrogel C possess optimal rheological properties for smooth application. High spreadability allows the formulation to conform to irregular wound surfaces, ensuring uniform contact and enhancing patient comfort by reducing shear stress on delicate tissue.^32^ Spreadability testing revealed an optimal spreading coefficient, ensuring uniform coverage and seamless contact when applied to biological tissues.

### 3.22. Swelling profile of hydrogels

**Fig. 17:**
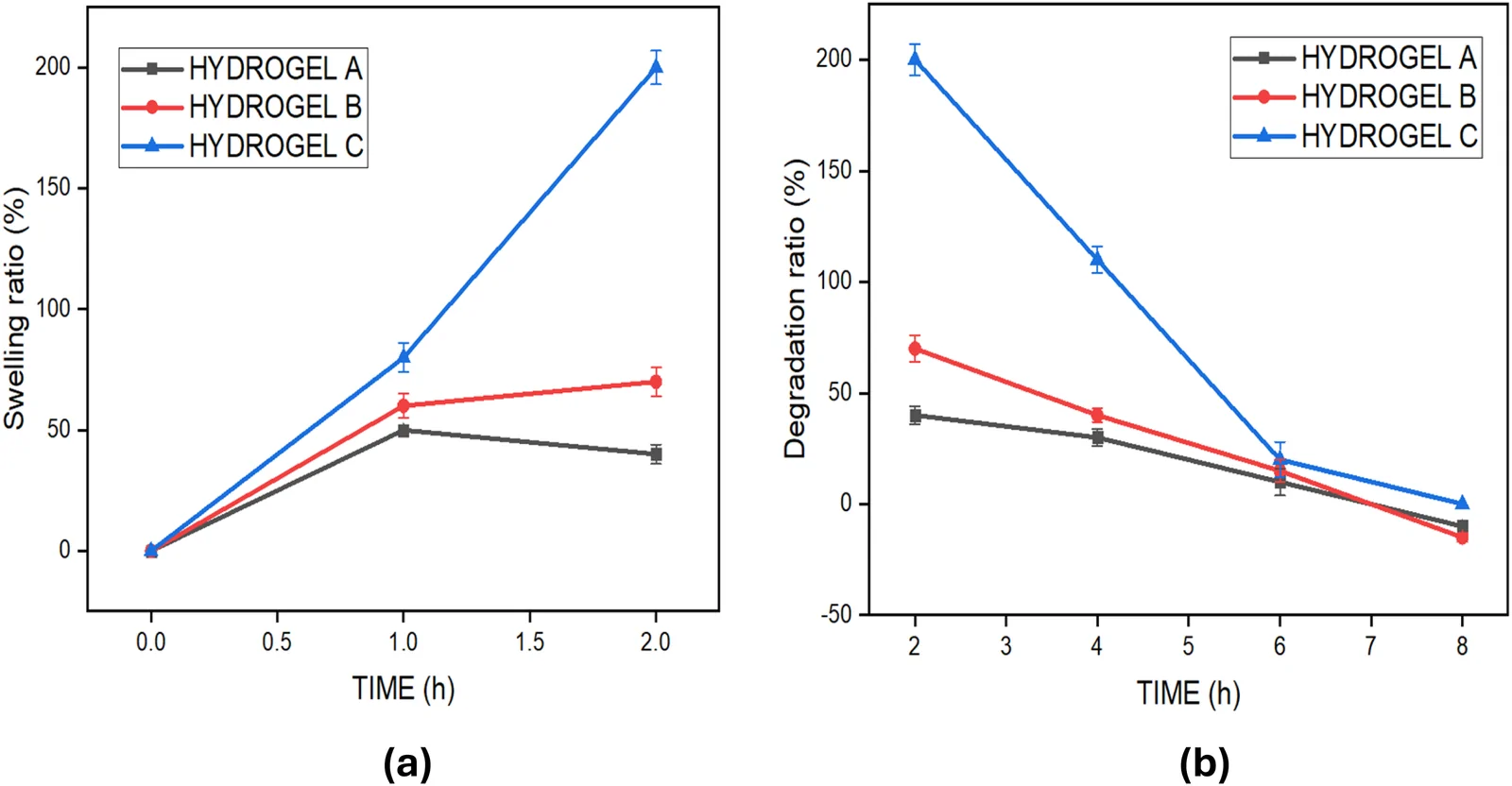
(a) Swelling of hydrogels, (b) degradation of hydrogels.

Fig. 17(a) shows the swelling kinetics of hydrogels A–C over time. All three formulations exhibit a time-dependent increase in swelling ratio, reaching equilibrium at 2 h. Hydrogel C demonstrates superior performance, achieving a maximum swelling ratio of approximately 200%. This significantly surpasses the capacities of hydrogel B (70%) and hydrogel A (40%). The high swelling ratio of hydrogel C is ideal for wound dressings, as it effectively absorbs exudate and maintains a hydrated environment necessary for healing. Consequently, hydrogel C possesses the most desirable swelling profile for effective wound management. The swelling kinetics of the hydrogel were evaluated in physiological buffer (PBS), of pH 7.4 at 37 °C, providing a baseline corresponding to healthy tissue interstitial fluid and systemic homeostasis. Under these conditions, the hydrogel demonstrated a stable swelling equilibrium and predictable fluid absorption without structural breakdown, maintaining matrix integrity for moisture retention. The hydrogels exhibited a rapid initial swelling phase followed by a stable equilibrium uptake, demonstrating an exceptional capacity to absorb physiological fluids.

### 3.23. Degradation profile of hydrogels

Fig. 17(b) displays the degradation test of hydrogels A–C over 8 h to evaluate stability at the wound site. Hydrogel C exhibits the fastest degradation rate, with its ratio dropping from 200% at 2 h to near 0% by 8 h. This rapid profile may suit dressings requiring frequent, non-invasive replacement or fast therapeutic release. In contrast, hydrogels A and B show more controlled degradation, dropping to approximately 10% and 0%, respectively. While hydrogel C is more dynamic, these results highlight its potential for specific applications needing quick removal and active delivery. The hydrolytic degradation of the hydrogel was monitored in physiological buffer of pH 7.4 at 37 °C, representing healthy tissue conditions. Under these environment parameters, the hydrogel exhibited predictable, gradual degradation kinetics without rapid dissolution, confirming that the crosslinked network retains structural stability over the target operational timeframe. Furthermore, degradation studies in demonstrated a controlled degradation profile, retaining structural integrity for up to 8 h before gradual matrix breakdown, matching the desired therapeutic window.

### 3.24. Colony count

**Fig. 18:**
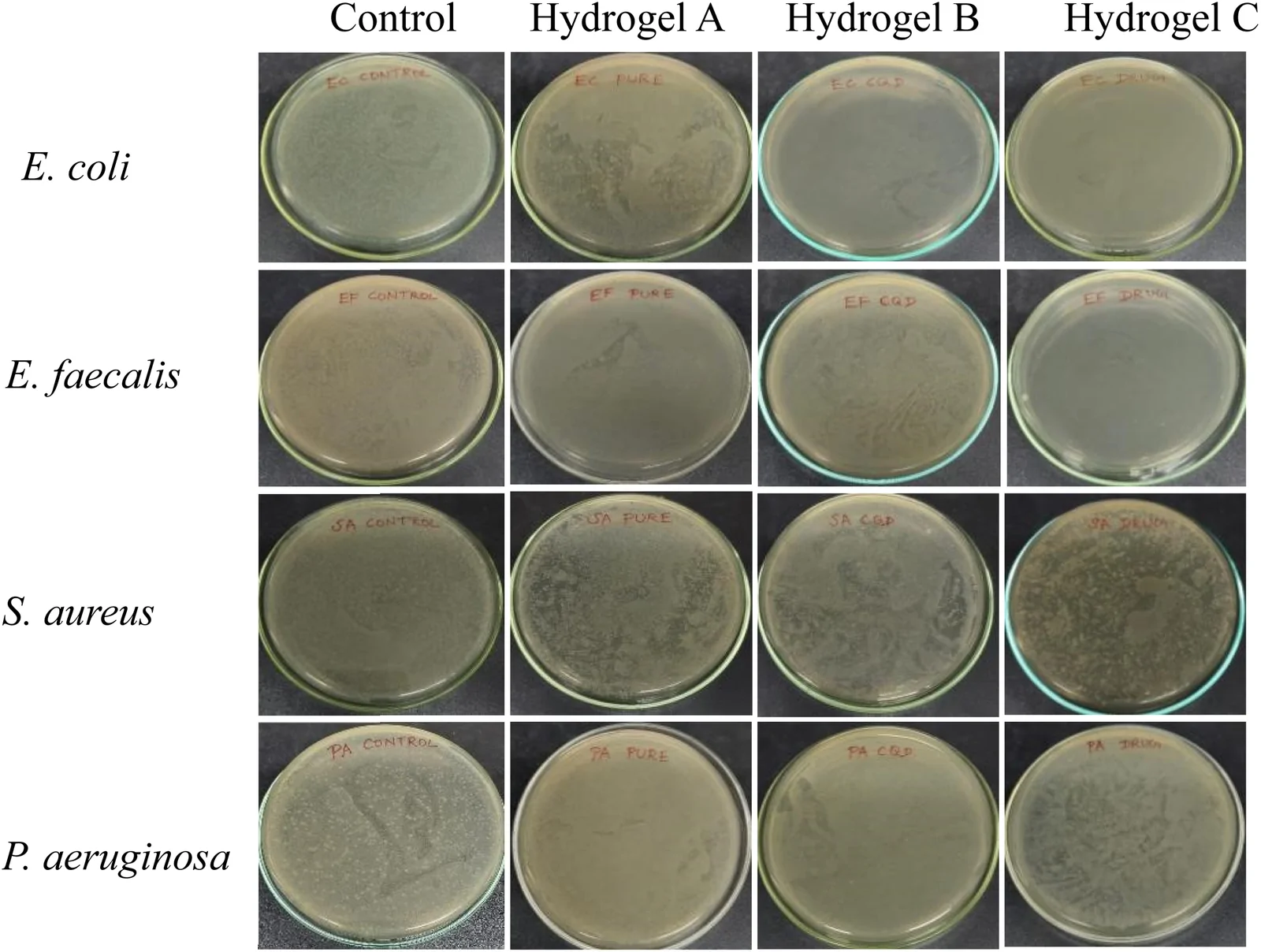
Colony count analysis of hydrogel A (pure), hydrogel B (CQD incorporated hydrogel), hydrogel C (CQD + quercetin incorporated hydrogel).

To quantitatively complement the bacterial growth patterns observed on the agar plates (Fig. 18), colony-forming unit (CFU mL^−1^) enumeration was conducted across all four bacterial strains (Table 4). The untreated control groups for *E. coli*, *E. faecalis*, *S. aureus*, and *P. aeruginosa* displayed confluent growth in the range of 1.60 × 10^6^ to 2.10 × 10^6^ CFU mL^−1^. The unfunctionalized hydrogel A exhibited modest baseline physical growth retardation, reducing bacterial counts by 17.5% to 21.4%. The incorporation of CQD's in hydrogel B significantly enhanced bactericidal activity, reducing bacterial viability to 0.80–0.95 × 10^6^ text CFU mL^−1^ (50.0% to 56.2% inhibition). This growth suppression is attributed to the surface functional groups on the CQDs inducing localized cell membrane disruption and ROS accumulation.

**Table 4:** Bacterial density (CFU mL^−1^) and percent reduction across hydrogel formulations against four bacterial strains

Pathogen	Evaluation parameter	Control	Hydrogel A	Hydrogel B	Hydrogel C
*E. coli*	CFU mL^−1^ (×10^6^)	1.85 ± 1 2	1.48 ± 0.10	0.85 ± 0.07	0.42 ± 0.04
	Inhibition (%)	0.0%	20.0%	54.1%	77.3%
*E. faecalis*	CFU mL^−1^ (×10^6^)	1.60 ± 0.10	1.32 ± 0.08	0.80 ± 0.06	0.45 ± 0.05
	Inhibition (%)	0.0%	17.5%	50.0%	71.9%
*S. aureus*	CFU mL^−1^ (×10^6^)	2.10 ± 0.15	1.65 ± 0.11	0.92 ± 0.08	0.48 ± 0.04
	Inhibition (%)	0.0%	21.4%	56.2%	77.1%
*P. aeruginosa*	CFU mL^−1^ (×10^6^)	1.95 ± 0.14	1.58 ± 0.12	0.95 ± 0.07	0.51 ± 0.05
	Inhibition (%)	0.0%	19.0%	51.3%	73.8%

The optimized hydrogel C formulation demonstrated the highest antimicrobial efficacy, achieving 71.9% to 77.3% bacterial reduction across both Gram-positive and Gram-negative pathogens. These quantitative metrics validate the visual growth attenuation observed in Fig. 18, confirming effective, concentration-dependent bacterial suppression while maintaining matrix integrity. Hydrogel C, loaded with both quercetin and CQDs, achieves the most substantial suppression of bacterial colonies across all species. This synergistic effect stems from the combination of the hydrogel's physical barrier, CQD mediated damage, and controlled drug release.^40^

### 3.25. MTT assay

**Fig. 19:**
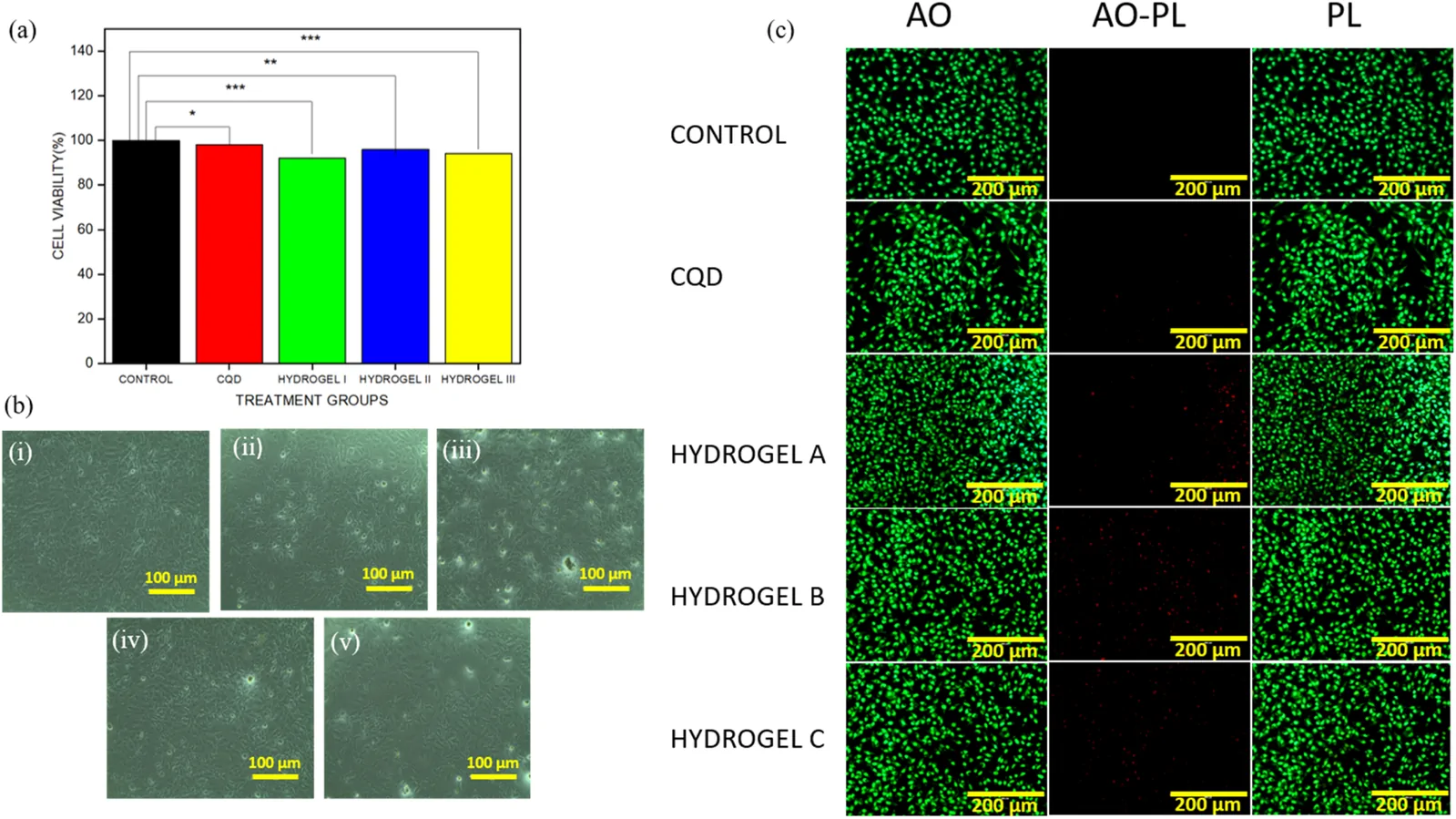
(a) Cell viability analysis of the hydrogels against NIH3t3 fibroblasts. (b) Optical image of NIH3t3 fibroblasts cells after treating them with hydrogels. (i) Control (ii) CQD (iii) hydrogel I(iv) hydrogel II(v) hydrogel III. (c) Cell viability (live/dead).

The biocompatibility of the synthesized hydrogels was successfully confirmed using the MTT assay with NIH/3T3 fibroblast cells, in Fig. 19(a) demonstrating their high potential for wound healing applications. All tested hydrogel formulations (A–C) and the component CQD exhibits excellent cell viability, consistently staying above 90% when compared to the untreated control (100%). Specifically, hydrogel B and hydrogel C shows cell viability near 95–96%, establishing them as the most compatible materials, as their results were statistically indistinguishable from the control. The optical image presented in Fig. 19(b) illustrates the appearance of NIH/3T3 fibroblast cells following exposure to the hydrogels which reveals that the hydrogels had no detrimental effect on the normal morphology of the cells, confirms their non-cytotoxic nature. The overall impressive performance of these formulations suggests they can be safely utilized as wound dressings without compromising cell health at the treatment site.

### 3.26. Live dead assay

To provide further confirmation of the biocompatibility, a live/dead viability assay is conducted on the control, CQD and hydrogel A–C under light illumination, allowing for a visual assessment of cell integrity. In this procedure, propidium iodide (PI) was used as a fluorescent probe to mark dead cells (red fluorescence), while fluorescein isothiocyanate (FITC) was employed to indicate live cells (green fluorescence). The resulting microscopic image, detailed in Fig. 19(c) which compares the live/dead images of cells treated with the hydrogels against the untreated control. Complementing qualitative microscopic observations (Fig. 19), quantitative live/dead cell enumeration was performed to evaluate the cytocompatibility of the hydrogel matrices (Table 5).

**Table 5:** Quantitative cell viability and dead cell percentages of hydrogel formulations using AO/PI staining (*n* = 3)

Treatment group	Viability (%)	Dead cells (%)
Control	99.4 ± 0.3%	0.6 ± 0.3%
CQD	98.8 ± 0.4%	1.2 ± 0.4%
Hydrogel I	96.4 ± 0.6%	3.6 ± 0.6%
Hydrogel II	95.5 ± 0.7%	4.5 ± 0.7%
Hydrogel III	94.5 ± 0.8%	5.5 ± 0.8%

The untreated control and pure CQD groups displayed high viability percentages of 99.4 ± 0.3% and 98.8 ± 0.4%, respectively, confirming the baseline non-toxicity of the CQDs. Upon incorporating CQDs and drug molecules into the polymer matrix, hydrogels A–C maintained excellent cell survival rates of 96.4 ± 0.6%, 95.5 ± 0.7%, and 94.5 ± 0.8%, respectively. The negligible proportion of dead cells (red fluorescence, <5.5%) demonstrates that the composite hydrogel platform provides a highly biocompatible microenvironment suitable for tissue engineering and wound dressing applications. Therefore, the results demonstrate that CQD, hydrogels A–C possess excellent biocompatibility, making them highly appropriate for use as wound dressing materials.

### 3.27. Animal studies

**Fig. 20:**
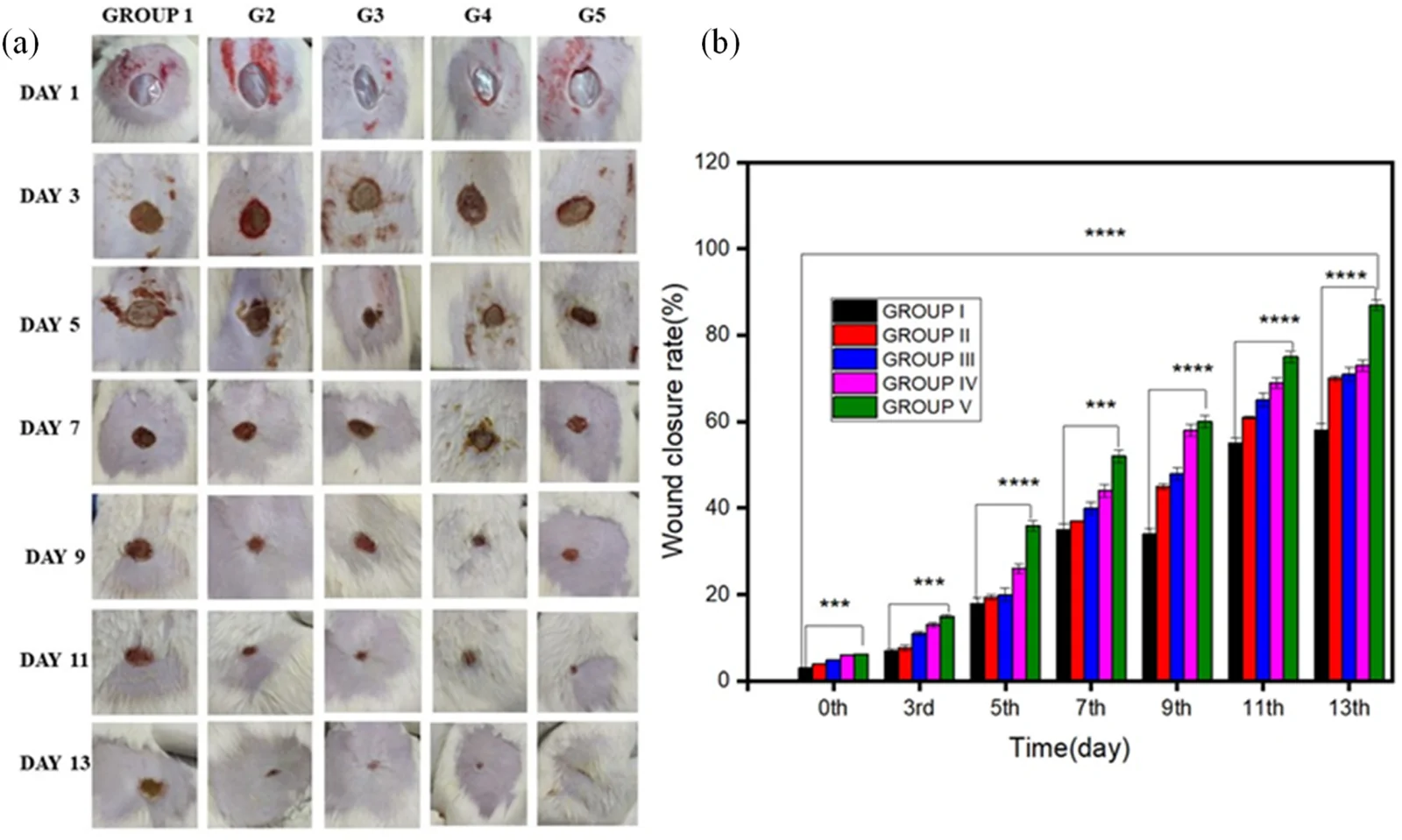
(a) Wound healing in albino Wistar rats, (b) wound closure rate of hydrogels.

**Fig. 21:**
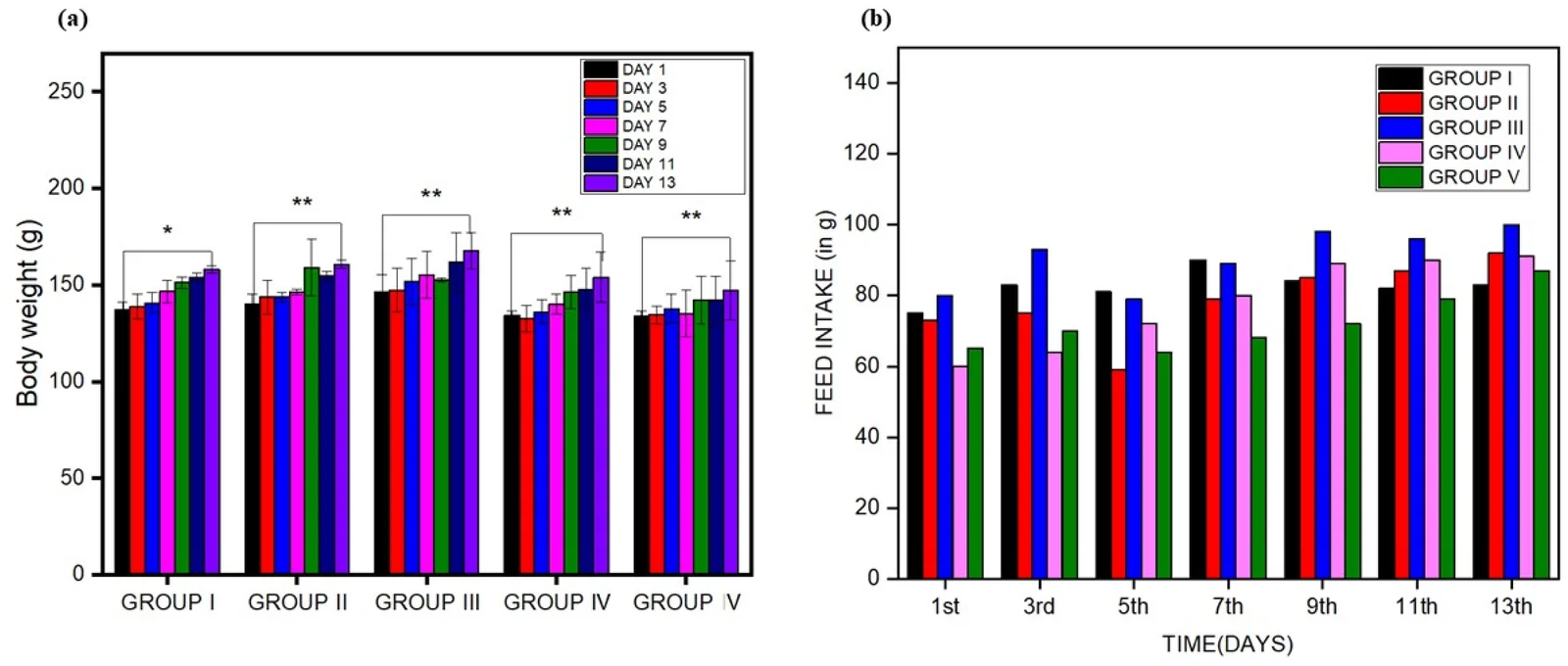
(a) Body weight of the experimental animals, (b) feed intake of albino Wistar rats.

**Fig. 22:**
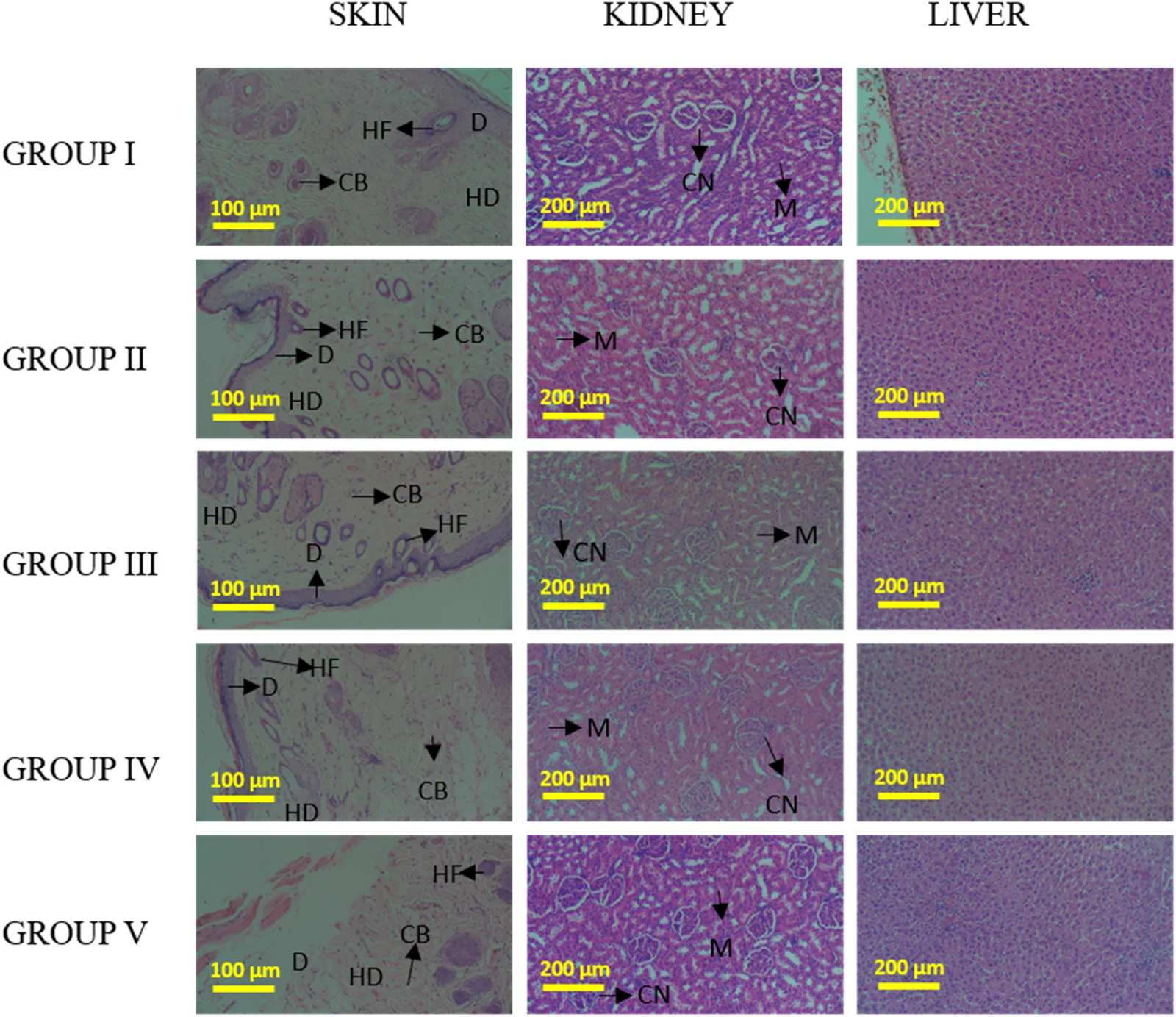
Histopathological analysis of skin (dermis (D), hypodermis (HD), hair follicle (HF), collagen bundle (CB)), kidney (cortical nephron (CN), medulla (M)) and liver.

*In vivo* wound healing experiments are conducted on albino Wistar mice to assess the therapeutic potential of developed hydrogel formulations.^41^ Group I (control) consists of untreated wounds, while Group II (standard) is treated with a commercial ointment (Hydra-Heal). The remaining groups receive specific formulations: Group III (hydrogel A) contains AV extract; Group IV (hydrogel B) incorporates CQDs; and Group V (hydrogel C) includes both CQDs and quercetin. Formulations are applied topically once daily for 13 days. Wound progression is monitored by tracing margins onto transparent sheets to quantify contraction (Fig. 20(a)). Wound closure rates in Fig. 20(b) show a progressive increase in healing. By day 13, the closure rates are: Group I (58%), Group II (70%), Group III (71%), Group IV (73%), and Group V (89%). Group V (hydrogel C) achieves the highest rate, significantly outperforming the standard treatment and other formulations. This confirms that hydrogel C effectively accelerates wound repair. On day 1, all groups exhibit inflammation and necrosis typical of initial injury. By day 3, Group V demonstrates scab formation and early signs of contraction, whereas wounds in Groups II, III, and IV still resemble the initial stage. By day 6, reduced swelling and new hair growth are evident in Groups II and V, while the control shows negligible progress. By day 9, wounds in Group V display substantial size reduction, collagen deposition, and epidermal reconstruction. In contrast, Groups III and IV exhibit slower regeneration, and Group I remains inflamed. By day 12, Group V shows almost complete wound closure with restored dermal architecture and dense hair growth, closely resembling normal skin. Quantitative analysis reveals that wound contraction is significantly higher in Group V compared to the control (*p* < 0.001). This superior efficacy is attributed to the synergistic effects of CQDs and quercetin, where quercetin's anti-inflammatory activity reduces scar formation and accelerates tissue repair. Body weight analysis (Fig. 21(a)) reveals a gradual, consistent increase across all groups, indicating that the treatments do not adversely affect general health. Similarly, all five groups exhibit stable feed consumption (Fig. 21(b)), typically fluctuating between 60 and 100 grams. This sustained feeding behavior suggests that neither surgical wounds nor the hydrogel administrations induce anorexia or significant systemic toxicity. Histopathological evaluation (Fig. 22) of skin tissues demonstrates enhanced repair in treated groups compared to controls. Hydrogel C shows the highest collagen formation throughout the dermal layer, accompanied by well-defined re-epithelialization. The dense organization of fiber indicates active tissue remodeling. In untreated controls, structural restoration is poor and slow. Similarly, kidney samples in Fig. 22. Shows preserved glomerular and tubular structures in all groups. The cortical nephron and medulla displayed intact cellular morphology without evidence of degeneration, tubular dilation, or interstitial inflammation. These observations collectively indicate that the administered hydrogels are biocompatible and do not elicit effects on major organs while effectively supporting skin tissue regeneration. Liver sections from control and hydrogel-treated rats exhibited comparable architecture in Fig. 22, with hepatocytes arranged in normal cords and no signs of necrosis, inflammation, or vascular congestion. The absence of pathological alterations across all treatment groups confirms that the hydrogels did not induce hepatic toxicity or functional disturbance.

The present study demonstrates that the incorporation of CQDs and quercetin into AV/CMC hydrogel significantly enhances antimicrobial efficacy and wound healing performance. Structural characterization confirms the successful synthesis of functionalized CQDs with surface-active groups that facilitate interaction with bacterial membranes. The hydrogel system exhibits favourable physicochemical properties, including optimal swelling, spreadability, and injectability, which collectively support a moist and protective wound environment. Antibacterial assays reveal a clear concentration-dependent inhibition of both Gram-positive and Gram-negative pathogens, accompanied by reduced biofilm formation, EPS production. Mechanistically, CQDs exert antimicrobial action through multiple pathways. They induce excessive intracellular ROS, leading to lipid peroxidation, protein oxidation, and membrane destabilization. Simultaneously, their nanoscale size enables direct interaction with bacterial cell walls, causing permeability loss and leakage of intracellular contents. Importantly, CQDs interfere with quorum sensing (QS) by disrupting signalling molecules such as acyl-homoserine lactones (AHLs), thereby inhibiting coordinated gene expression responsible for biofilm maturation and virulence.

Quercetin further amplifies this effect by acting as a quorum sensing inhibitor (QSI). It competitively binds to QS regulatory proteins and suppresses signal transduction pathways, reducing EPS synthesis and bacterial communication. Additionally, its antioxidant and anti-inflammatory properties modulate the wound microenvironment, promoting fibroblast proliferation and collagen deposition. The synergistic integration of CQDs and quercetin in hydrogel C results in superior bacterial inhibition and accelerated wound closure (89% by day 13). This combined mechanism highlights a multifunctional strategy targeting both bacterial survival and communication, making the developed hydrogel a promising candidate for advanced wound care applications.

## 4 Conclusion

CQDs displays significant antibacterial efficacy against bacteria commonly present on wound surface, as demonstrated by *in vitro* experiments. Furthermore, hydrogels A–C exhibits potent antimicrobial characteristics against typical wound pathogens. Their capacity to swell and absorb large amounts of fluid exudate is an additional functional benefit. In cell culture tests, these hydrogels were shown to be non-toxic and actively supported cell viability, growth, and mobility. Crucially, the wound healing capabilities were confirmed *in vivo* using Albino Wistar rats, proving that hydrogels A–C are biocompatible materials that accelerate the healing of wounds with hydrogel C aiding in effective wound closures. These findings agree well with recent developments in multifunctional wound dressings, where nanoparticle-driven membrane disruption works synergistically with natural polyphenols. The presence of CQDs strengthens the polymer matrix while quercetin scavenges free radicals to accelerate tissue recovery. Overall, this nanocomposite hydrogel offers a balanced, infection-resistant microenvironment ideal for modern chronic wound management.^46^

## Ethical statement

All animal experiments were conducted in strict accordance with the guidelines established by the Committee for the Purpose of Control and Supervision of Experiments on Animals (CPCSEA), Government of India, and were approved by the Institutional Animal Ethics Committee (IAEC) of SRM Institute of Science and Technology, Kattankulathur, Tamil Nadu, India. All experimental procedures were designed and conducted in compliance with the 3R principles- replacement, reduction, and refinement- to minimize animal use and ensure humane treatment throughout the study. Animals were housed under standard laboratory conditions at 22 ± 2 °C with a 12 h light/dark cycle and provided with standard rodent chow and water ad libitum. All surgical interventions were performed under appropriate anesthesia, and animals were monitored postoperatively for signs of pain or distress. Humane endpoints were defined prior to commencement of the study. All personnel involved in animal handling were trained and certified in accordance with CPCSEA requirements.

## Author contributions

KB- formal analysis, data curation., writing – original draft., MSM- formal analysis, writing – original draft, visualization, and data curation., SRS- writing – review & editing., BP- formal analysis; validation; visualization., DVG-conceptualization; supervision; validation; writing – review & editing.

## Conflicts of interest

The authors have no conflicts to disclose.

## Data Availability

The data that support the findings of this study are available from the corresponding author upon reasonable request.
